# Supplementary material for: learnMSA: learning and aligning large protein families
Source: Gigascience. 2022 Nov 18;11:giac104. doi: 10.1093/gigascience/giac104 (PMC9673500; doi:10.1093/gigascience/giac104)
Supplement: giac104_GIGA-D-22-00144_Revision_2 [file giac104_giga-d-22-00144_revision_2.pdf]

GigaScience

learnMSA: Learning and Aligning Large Protein Families

--Manuscript Draft--

**Manuscript Number:**	GIGA-D-22-00144R2
**Full Title:**	learnMSA: Learning and Aligning Large Protein Families
**Article Type:**	Technical Note
**Funding Information:**	
**Abstract:**	Background: The alignment of large numbers of protein sequences is a challenging task and its importance grows rapidly along with the size of biological datasets. State-of-the-art algorithms have a tendency to produce less accurate alignments with an increasing number of sequences. This is a fundamental problem since many downstream tasks rely on accurate alignments.<br>Results: We present learnMSA, a novel statistical learning approach of profile hidden Markov models (pHMMs) based on batch gradient descent. Fundamentally different from popular aligners, we fit a custom recurrent neural network architecture for (p)HMMs to potentially millions of sequences with respect to a maximum a posteriori objective and decode an alignment. We rely on automatic differentiation of the log-likelihood and thus, our approach is different from existing HMM training algorithms like Baum–Welch. Our method does not involve progressive, regressive or divide-and-conquer heuristics. We use uniform batch sampling to adapt to large datasets in linear time without the requirement of a tree. When tested on ultra-large protein families with up to 3.5 million sequences, learnMSA is both more accurate and faster than state-of-the-art tools. On the established benchmarks HomFam and BaliFam with smaller sequence sets it matches state-of-the-art performance. All experiments where done on a standard workstation with a GPU.<br>Conclusions: Our results show that learnMSA does not share the counter-intuitive drawback of many popular heuristic aligners which can substantially lose accuracy when many additional homologs are input. LearnMSA is a future-proof framework for large alignments with many opportunities for further improvements.
**Corresponding Author:**	Mario Stanke, Dr. rer. nat.<br>University Greifswald Faculty of Mathematics and Natural Sciences: Universitat Greifswald Mathematisch-Naturwissenschaftliche Fakultat<br>Greifswald, MV GERMANY
**Corresponding Author Secondary Information:**	
**Corresponding Author's Institution:**	University Greifswald Faculty of Mathematics and Natural Sciences: Universitat Greifswald Mathematisch-Naturwissenschaftliche Fakultat
**Corresponding Author's Secondary Institution:**	
**First Author:**	Felix Becker, M.Sc.
**First Author Secondary Information:**	
**Order of Authors:**	Felix Becker, M.Sc.
	Mario Stanke, Dr. rer. nat.
**Order of Authors Secondary Information:**	
**Response to Reviewers:**	We added the citation to the GigaDB dataset.
**Additional Information:**	
**Question**	**Response**
Are you submitting this manuscript to a special series or article collection?	No
**Experimental design and statistics**	Yes

Powered by Editorial Manager® and ProduXion Manager® from Aries Systems Corporation

Full details of the experimental design and statistical methods used should be given in the Methods section, as detailed in our Minimum Standards Reporting Checklist. Information essential to interpreting the data presented should be made available in the figure legends.<br><br>Have you included all the information requested in your manuscript?	
**Resources**<br><br>A description of all resources used, including antibodies, cell lines, animals and software tools, with enough information to allow them to be uniquely identified, should be included in the Methods section. Authors are strongly encouraged to cite Research Resource Identifiers (RRIDs) for antibodies, model organisms and tools, where possible.<br><br>Have you included the information requested as detailed in our Minimum Standards Reporting Checklist?	Yes
**Availability of data and materials**<br><br>All datasets and code on which the conclusions of the paper rely must be either included in your submission or deposited in publicly available repositories (where available and ethically appropriate), referencing such data using a unique identifier in the references and in the "Availability of Data and Materials" section of your manuscript.<br><br>Have you have met the above requirement as detailed in our Minimum Standards Reporting Checklist?	Yes

Manuscript

Click here to access/download;Manuscript;MSA_HMM_Paper.pdf

OXFORD

(GIGA)^n SCIENCE

GigaScience, 2017, 1–13

doi: xx.xxxx/xxxx
Manuscript in Preparation
Paper

PAPER

learnMSA: Learning and Aligning Large Protein Families

Felix Becker[1,*] and Mario Stanke[1,*]

[1]Institute of Mathematics and Computer Science, University of Greifswald, Germany

Correspondence: *felix.becker@uni-greifswald.de; mario.stanke@uni-greifswald.de

Abstract

Background: The alignment of large numbers of protein sequences is a challenging task and its importance grows rapidly along with the size of biological datasets. State-of-the-art algorithms have a tendency to produce less accurate alignments with an increasing number of sequences. This is a fundamental problem since many downstream tasks rely on accurate alignments.
Results: We present learnMSA, a novel statistical learning approach of profile hidden Markov models (pHMMs) based on batch gradient descent. Fundamentally different from popular aligners, we fit a custom recurrent neural network architecture for (p)HMMs to potentially millions of sequences with respect to a maximum a posteriori objective and decode an alignment. We rely on automatic differentiation of the log-likelihood and thus, our approach is different from existing HMM training algorithms like Baum-Welch. Our method does not involve progressive, regressive or divide-and-conquer heuristics. We use uniform batch sampling to adapt to large datasets in linear time without the requirement of a tree. When tested on ultra-large protein families with up to 3.5 million sequences, learnMSA is both more accurate and faster than state-of-the-art tools. On the established benchmarks HomFam and BaliFam with smaller sequence sets it matches state-of-the-art performance. All experiments where done on a standard workstation with a GPU.
Conclusions: Our results show that learnMSA does not share the counter-intuitive drawback of many popular heuristic aligners which can substantially lose accuracy when many additional homologs are input. LearnMSA is a future-proof framework for large alignments with many opportunities for further improvements.

Key words: profile hidden Markov model, multiple sequence alignment, machine learning

Background

Profile hidden Markov models (pHMMs) are probabilistic models for protein families. One of their applications is remote homology search in large databases [1, 2]. Typically, an existing multiple sequence alignment (MSA) is turned into a pHMM, however, pHMMs can also be trained on unaligned sequences and a MSA can be decoded from the learned model [3, 4, 5]. The training of pHMMs using the Baum-Welch algorithm was originally applied 'with hand-holding' to selected protein families [3], which required a human to decide between specific architectures, e.g. for modeling a domain as opposed to an entire protein. Advantages of the statistical learning approach over traditional aligners are a consistent probabilistic background for position-specific gap penalties and that both training and decoding are linear in the number of sequences. However, profile HMM training has never been popular as a general-purpose alignment method since *tabula rasa* learning is challenging. Apart from the model architecture being problem dependent, another common issue is that algorithms may get stuck at local optima in the parameter space. Simulated annealing [4] and particle swarm optimization [6, 7] could further improve upon Baum-Welch in this regard, but never resulted in applicable tools comparable to modern state-of-the-art aligners. Gradient descent methods like the popular Adam algorithm [8] are an hitherto entirely unexplored class of algorithms for HMM

Compiled on: October 4, 2022.
Draft manuscript prepared by the author.

training with increasing relevance in the advent of automatic differentiation [9].

Established tools that construct MSAs are either unfit for large numbers of sequences or their accuracy decreases when the number of aligned sequences grows large [10, 11]. This effect is particularly present for progressive algorithms, which rely on a guide tree that dictates the order of the sequences to be aligned, by greedily starting with closely related ones. One drawback of this approach is the inability to revert gaps. Early errors accumulate when more and more sequences are added.

One way to revert incorrect gaps is iterative refinement, where intermediate alignments guide the construction of subsequent ones [12]. Although iterative refinement strategies can improve accuracy on moderate sequence numbers, they are unsuitable for large numbers of sequences from a computational perspective. For example, MAFFT G-INS-i produces very accurate alignments, but is slow and memory-hungry due to an all-to-all pairwise alignment stage. MAFFT-Sparsecore applies MAFFT G-INS-i to a small set of core sequences and progressively added the remaining sequences thereafter [13]. This strategy is suitable to scale up iterative refinement to large sequence numbers, but biases in the core sequences have to be avoided by choosing them as diverse as possible.

Divide-and-conquer strategies like PASTA [14] and MAGUS [15] first construct subalignments on relatively small subsets of the sequences and merge them thereafter. MAGUS uses a Graph Clustering Merger for the latter stage. Recently, MAGUS was updated to support recursion for ultra-large datasets [16]. Another technique with improved accuracy is the regressive method which starts to align sequences containing the most *dissimilar* ones first and merges subalignments by using an overlapping sequence [10]. Divide-and-conquer strategies have enabled the execution of slow but accurate algorithms like MAFFT G-INS-i on large datasets and improved accuracy compared to progressive strategies [10, 15]. However, they are still heuristics that ignore everything but a subset at first and are prone to errors in their merging steps.

Lastly, UPP [17] is related to our method by the fact that it also uses a pHMM (or an ensemble of pHMMs) to represent MSAs. However, UPP does not train a model on unaligned sequences. Instead, it first constructs a backbone MSA on a subset of the sequences using tree-guided PASTA in order to estimate the HMM parameters. Afterwards, it adds the remaining sequences using the HMM. UPP has shown good performance in the presence of high sequence length heterogeneity.

All mentioned MSA algorithms rely on accurate guide trees and tree construction often becomes the computational bottleneck. Clustal Omega [11] uses the mbed method to construct a tree. A faster but less accurate alternative is MAFFT-PartTree [18] and another popular algorithm is FastTree [19]. A slow but very accurate tree construction algorithm based on all-to-all pairwise alignments is used in the G-INS-i option of MAFFT [12]. The bottom line is the constant need to balance quality and speed when constructing trees.

To date, deep learning is not commonly used for multiple sequence alignment and if it is, its function is usually supplementary, e.g. by optimizing the order of progressive alignment with reinforcement learning [20] or employing a decision making model to select from different strategies in a MSA pipeline [21]. While some proof of concepts exist, the respective software is not feasible for large numbers of sequences, generally not optimized (stated by the authors) or not available at all. For pairwise alignment the traditional dynamic programming framework can be supplemented by reinforcement learning [22] or deep models inspired by recent advances in natural language processing improving accuracy on remote homologs [23]. While deep learning is currently usually not used for their construction, MSAs are, however, a popular input for end-to-end machine learning methods that solve downstream tasks [24, 25, 26, 27].

Our proposed aligner learnMSA is based on automated statistical learning of a pHMM with gradient descent. It does not require a tree and has a linear asymptotic runtime in the number of sequences which is faster than most tree algorithms. No progressive, regressive or divide-and-conquer heuristic is used. Therefore, we avoid heuristic-based errors when merging subalignments or progressively adding sequences. We provide a more robust framework for (ultra-)large MSAs without the counter-intuitive drawback of loosing accuracy when many additional homologs are input.

We begin with the description of the underlying model and a batch-wise variant of the forward algorithm that plays a central role during parameter training. We empirically show the suitability of learnMSA by testing it on ultra-large protein families from Pfam [28] with up to 3.5 million sequences as well as the established biological benchmarks HomFam and BaliFam.

Methods

Model

Profile hidden Markov models are well known probabilistic models of sequence consensus. When used to model a protein family, the aim is to define a probability distribution over the space of all possible protein sequences such that member sequences of the family have large probabilities. The resulting statistical model can be used for database searches [1] and MSA construction [3].

In a pHMM, a linear chain of match states represents the consensus sequence of the family in question. Insertions and deletions with respect to the consensus are modeled by position-specific states and transitions. See Figure 1-A for an illustration of the pHMM.

In addition to the standard pHMM architecture, we deploy an augmented model following HMMER's 'Plan7' [29, 30] (orange states and transitions in Figure 1-A). The HMM parameters are learned from unaligned protein sequences. In contrast to previous approaches, our method also learns the additional 'Plan7' parameters jointly with the pHMM core model. Previously, HMMER used predefined value sets for different alignment modes (local or global, unihit or multihit) [30]. Here, we automatically learn the correct alignment mode jointly with the core pHMM starting *tabula rasa*. We have special states for the left (L) and right (R) flank of the model. Initialization and regularization of the flanking states differ from ordinary insertion states I_i (see section 'Training'). Moreover, the augmented model allows multihit alignments, i.e. sequences may contain repeats of a single domain motif by looping backwards. The state C models any unannotated region between two domain hits and must be visited to jump from the end state E back to the start state S. The model further handles sequence length heterogeneity (fragmentary sequences) through entry- and exit-probabilities from S into the consensus and, respectively, from the consensus to E. Note that since version 2, HMMER uses a trick to achieve a uniform distribution over all possible pairs of entry- and exit points into the core model [30]. Here, we follow the older construction with explicit entry- and exit-probabilities, however, they are now data-dependent instead of *ad hoc*.

The set of all transition- and emission parameters is learned from data with careful initialization and under the use of Dirichlet priors (see section 'Training'). In general, we have one trainable parameter for each possible state transition and in case of the emissions one parameter per match state and amino acid. There are exceptions: Insertion- and flanking

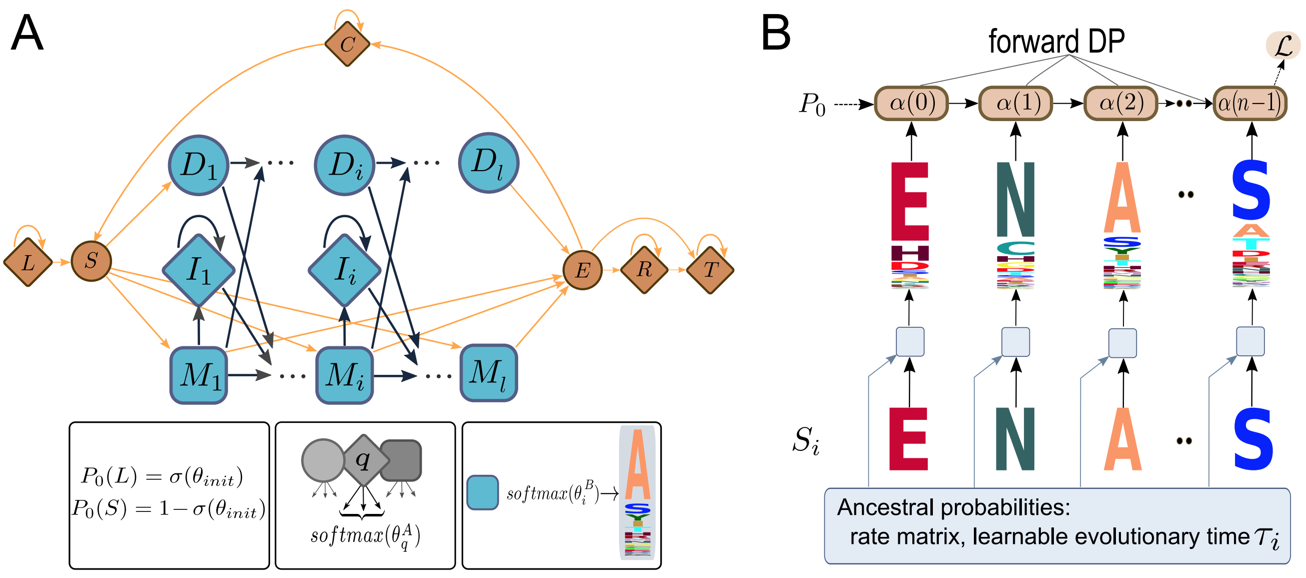

Figure 1. A: LearnMSAs underlying pHMM based on HMMER's 'Plan7' model. For the transition (emission) distributions, unconstrained learnable parameter matrices θ^A (θ^B) are transformed by softmaxes over the outgoing edges of a state or the amino acid alphabet respectively. Squares indicate match states, diamonds are insertions and circles are silent states (either delete states or the start- and end-state). In contrast to previous approaches, we also learn transition probabilities augmenting the core model (orange). **B:** Sketch of a recurrent neural network architecture with a HMM-Cell that implements the forward recursion. The first layer at the bottom computes ancestral distributions of amino acids for a sequence S_i using a rate matrix and an evolutionary time τ_i that is learned jointly with the HMM parameters.

states use a fixed background emission distribution that is not optimized. The self-loop (and respectively exit) probabilities for the flanking states L, R and C are tied to prevent a bias towards one of the sides. Delete states (as well as the domain start- and end-states S and E) are silent, i.e. they have no emission distribution and do not not advance the position in the observed sequence.

A pHMM can be parameterized by two probability matrices for transitions and emissions and an initial state distribution. Let Q be the set of all states and A be the stochastic $|Q| \times |Q|$ matrix of state transitions. Observe that for pHMMs, this matrix is very sparse. We call the number of match states in a model its *length* l. Let $Q' := Q \setminus \{D_1, \ldots, D_l, S, E\}$ denote the set of all emitting states. Let B be the $|Q'| \times 25$ emission matrix, that is constructed by concatenating l learnable emission distributions of the match states with background distributions for all insertions and the flanks. The second dimension of B corresponds to the 20 standard amino acids, plus Selenocysteine, Pyrrolysine and the ambiguous codes X, B and Z. The terminal symbol (26-th letter) has an implicit probability of 0 at all states except T.

In order to apply gradient descent, we parameterize the model by unconstrained kernels θ^A and θ^B and enforce the probabilistic constraints that the rows of A and B sum up to 1 with a *softmax*-function defined on a real vector: $softmax(x)_i = \frac{e^{x_i}}{\sum_j e^{x_j}}$.

As seen in Figure 1-A the emission distribution of, for example, M_i is computed by $softmax(\theta_i^B)$, where $\theta_i^B = (\theta_A^{B_i}, \theta_R^{B_i}, \theta_N^{B_i}, \theta_D^{B_i}, \ldots)$ is the i-th row of θ^B. The matrix B is constructed from the kernel θ^B by using softmaxes to compute the match distributions over the amino acid alphabet.

The kernel θ^A is a collection of parameter vectors corresponding to different transition types that share the same initialization and prior. For example, we have $l-1$ parameters for the match-to-match transitions. The total number of allowed transitions in the model as shown in Figure 1-A is linear in l. The probability distribution of transitioning from – for example – match M_i to one of the 4 adjacent states M_{i+1}, I_i, D_{i+1} or E is calculated by constructing the vector $\theta_{M_i}^A = (\theta_{M_i,M_{i+1}}^A, \theta_{M_i,I_i}^A, \theta_{M_i,D_{i+1}}^A, \theta_{M_i,E}^A)$ and computing $softmax(\theta_{M_i}^A)$. We store A (or in fact, a matrix closely related to A as described in section 'Implicit model') in sparse matrix representation where illegal transitions are implicitly zero.

For the initial state distribution P_0, we use a simple parametrization by introducing a scalar θ_{init} that controls the probability of starting in the left flank. To this end, we define $p_{init} = \sigma(\theta_{init})$ where σ is the sigmoid function. The initial distribution is $P_0(L) = p_{init}$ and $P_0(S) = 1 - p_{init}$ and $P_0(q) = 0$ for $q \neq L, S$.

In the following, let $\theta = (\theta_{init}, \theta^A, \theta^B)$ denote the complete set of learnable parameters for the (augmented) pHMM.

Batch-wise forward algorithm

Assume for now that no silent states exist. For the pHMM as introduced in section 'Model', we will describe an equivalent implicit model without the silent states $D_1, \ldots, D_l, S$ and E in section 'Implicit model'. Consequently, $Q = Q'$ for now.

An unaligned protein sequence S can be described by a path π of hidden states in the pHMM. Under our assumption $\pi = \pi_0, \ldots, \pi_{n-1}$ and $S = s_0, \ldots, s_{n-1}$ have the same length n. The joint probability of observed and hidden sequence is $P(S, \pi) = P_0(\pi_0)P(s_0 \mid \pi_0)\prod_{i>0} P(\pi_i \mid \pi_{i-1})P(s_i \mid \pi_i)$, where the transition- and emission probabilities are computed as described in section 'Model' above.

The likelihood of a sequence is the sum of the joint probabilities over all possible hidden paths: $P(S) = \sum_\pi P(S, \pi)$ which is related to HMMER's forward score [30]. Intuitively, it describes how well a sequence fits to the consensus when considering all possible alignments. The likelihood can be efficiently computed with dynamic programming using either the forward- or the backward algorithm [31]. We present a batch-wise variant of the forward algorithm that plays a central role during parameter training of learnMSA.

The forward probabilities are $\alpha(i)_q := P(\pi_i = q, s_0, \ldots, s_i)$. The well-known dynamic programming recursion to compute

$\alpha(1), \ldots, \alpha(n-1)$ is

$$\alpha(i)_q = P(s_i \mid q) \sum_{q' \in Q} P(q \mid q')\alpha(i-1)_{q'} \quad (1)$$

with $\alpha(0)_q = P(s_0 \mid q)P_0(q)$.

Equation (1) lends itself to an efficient implementation for a batch of sequences of size b. Let the $b \times 25$ matrix $S^{(i)}$ denote the tuple of all i-th sequence positions in the batch, that is $S^{(i)}_j$ is an one-hot representation of the i-th residue of sequence j. We omit the implementation detail that for variable length sequences some positions might be terminal symbols here. In the following, we factor out a partial likelihood term in each forward step to allow an underflow-safe computation of the likelihood. The batch-wise forward recursion is:

$$\alpha'(i) = \begin{cases} S^{(i)}B^T \circ \frac{\alpha'(i-1)}{\mathcal{Z}(i-1)}A, & i > 0 \\ S^{(0)}B^T \circ P_0, & i = 0 \end{cases}$$
$$\mathcal{Z}(i) = \sum_{q \in Q} \alpha'(i)_q \quad (2)$$
$$\mathcal{L}(i) = \ln \mathcal{Z}(i).$$

where i is a sequence index, $\alpha'(i)$ are $b \times |Q|$ batches of scaled forward variables, $\circ$ denotes element-wise multiplication (with shape broadcasting, where required) and the matrix multiplication that involves A uses an efficient implementation that exploits the sparse representation. Observe that $\alpha(i) = \alpha'(i) \circ \prod_{i'=0}^{i-1} \mathcal{Z}(i')$.

The likelihood (for a single sequence) can eventually be computed as $P(S) = \sum_q \alpha(n-1)_q$. However, we prevent numerical underflow by equivalently using the partial log-likelihood values in Equation (2):

$$\ln P(S) = \sum_{i=0}^{n-1} \mathcal{L}(i). \quad (3)$$

Viterbi decoding

When we decode an alignment, we are interested in the hidden path of a sequence with maximum probability i.e. $\arg\max_\pi P(S, \pi)$. This can be computed efficiently using the Viterbi algorithm [31] which is closely related to the forward algorithm.

A Viterbi MSA can be constructed by aligning the most likely hidden sequences of all input sequences [3]. Currently, we leave insertions unaligned and left-adjusted except for the left flank which is right-adjusted. Moreover, if domain repeats occur, the i-th occurrences of the domain in multiple sequences respectively are currently aligned with each other. With both simplifications, we accept that we are in a slight disadvantage compared to state-of-the-art aligners which will align all residues globally.

Implicit model

Conventionally, the forward recursion for pHMMs is implemented in linear time per step by explicitly handling silent states (the deletes D_i, the starting state S and the ending state E) [32]. This requires a long-winded sequential computation of the forward variable for the delete states where $\alpha(i)_{D_j}$ depends on $\alpha(i)_{D_{j-1}}$. Here, we treat all silent states as implicit states, that is, internally we use an equivalent model that has only emitting states, by folding all transitions entering and leaving a silent state. That means all possible partial state paths that start and end in an emitting state and consist only of silent states else, are replaced by single transitions that have probability equal to the probability of the respective partial path. In detail, each partial path $M_i \to D_{i+1} \to \ldots D_{j-1} \to M_j$ for $j > i+1$ is replaced by an edge with probability

$$P(M_j \mid M_i) = P(D_{i+1} \mid M_i) \left(\prod_{i'=i+1}^{j-2} P(D_{i'+1} \mid D_{i'}) \right) P(M_j \mid D_{j-1}). \quad (4)$$

This changes the asymptotic runtime of the forward algorithm, because the number of possible transitions from each match state is not constant anymore. However, we can now implement Equation (2) by taking full advantage of modern (GPU-accelerated) computing frameworks. We found that given the typical length of a protein (our benchmarks contain sequences of length up to 800) the asymptotic downgrade is acceptable in the light of parallelism: We can compute all values of $\alpha(i)$ in parallel given $\alpha(i-1)$. In the batch-wise forward algorithm, the bottleneck is the matrix multiplication with the transition matrix which should use an efficient implementation that exploits sparseness.

Folding all edges adjacent to silent states is referred to as the implicit model, represented by a transition matrix A_{impl} replacing A from section ‘Model’. Note that A_{impl} is still very sparse. Transitions over the start state S and the end state E, i.e. deletions of initial or terminal parts, are handled analogously. Also note that empty, infinite silent loops through the model are not possible, because the unannotated segment state C is an insertion that emits at least one amino acid and can not be skipped.

Training

During training, learnMSA uses a recurrent neural network architecture with a pHMM cell that scans a batch of sequences column-of-residues-wise and successively applies Equation (2). This architecture is visualized in Figure 1-B with the addition of ‘Ancestral probabilities’ as described later. Given θ, the parameters of the model, the log-likelihood of a random batch of b sequences is

$$\mathcal{L}(\theta; S_1, \ldots, S_b) = \sum_{i=1}^{b} \ln P(S_i \mid \theta). \quad (5)$$

The general goal while successively observing random batches is to adjust θ such that $\mathcal{L}$ increases over time. In practice, we minimize a loss function related to $\mathcal{L}$ that also incorporates prior knowledge about proteins.

Existing optimization algorithms like Baum-Welch [3] or simulated annealing [4] avoid using gradients of $\mathcal{L}$ and use the forward-backward algorithm for parameter updates instead. An advantage of learnMSA is the possibility to optimize the HMM jointly with other layers. Currently, we demonstrate this as described in section ‘Ancestral probabilities’, but a broader field opens up in this direction as discussed later. Gradient based optimization can also be applied to objectives that are not based on likelihood, for instance the discrimination or classification of (sub)families [33]. Traditional HMM learning algorithms are not used for online learning although such variants exist [5]. Typically, they require more technical work to include priors than our gradient based approach. None of the methods can guarantee globally optimal results. However, learnMSA can make use of the advancing optimization toolbox for machine learning problems based on automatic differentiation [9].

Table 1. Dirichlet parameters for the core pHMM transition distributions estimated from Pfam HMMs

α	match	insert	delete
match	40.59	0.96	0.68
insert	26.75	23.32	-
delete	37.79	-	25.15

Maximum a posteriori loss

Models found by maximizing $\mathcal{L}$ might generalize weakly. This is especially true if the number of training sequences m is low. Our experiments will mainly focus on cases where m is large (i.e. 10.000 to millions of sequences). However, we can still have overfitting problems. Domain motifs of subfamilies might be underrepresented in the sequence set leading to a skewed model. Moreover, we might end up with a result that fits the data well but is not biologically plausible (e.g. a model that allows very long insertions or many gap openings). A maximum a posteriori estimate attempts to fit the data while at the same time penalizing unplausible models [3]. In this sense, we define our loss function as:

$$\ell(\theta; S_1, \ldots, S_B) = -\frac{1}{b}\mathcal{L}(\theta; S_1, \ldots, S_B) - \frac{1}{m}\ln(\rho(\theta)). \quad (6)$$

The loss ℓ has a foundation in Bayesian statistics. The first term is the log-likelihood per sequence averaged over a batch of sequences. Usually we choose $b < m$ and consequently perform stochastic gradient descent. This allows us to rapidly train models even on millions of homologous sequences. We use random uniform batch sampling. The second term is the prior density i.e. ρ is a function that rewards plausible models. We normalize by $\frac{1}{m}$ to make the estimate consistent. The effect of the prior is reduced proportional to the number of training sequences. This is particularly important because we use a general (i.e. family-agnostic) prior that should work over the full range of dataset sizes. Following conventional standards [3], we use Dirichlet densities [34, 35] over the different types of transition distributions and the match emissions.

To reduce the total number of hyperparameters that have to be set by hand, we salvaged as much general-purpose information as possible from Pfam HMMs. For the core model probabilities, we took over 3 million example transition distributions and maximized the likelihoods of 3 Dirichlets: One for matches, insertions and deletes respectively (see Table 1).

For the emissions, we tested Dirichlet mixtures with different component counts (1, 9, 32, 64, 128, 512) which we trained on the match emission distributions of Pfam HMMs, but found that for large sequence counts, a single Dirichlet density (i.e. a mixture with one component) is enough. The expectation of this Dirichlet distribution is also used to initialize the match emissions as well as the (fixed) insertion emissions and the flanks.

As described earlier, we optimize the transition probabilities for flanking states, domain multihits and the entry- and exit-probabilities jointly with the core model. We found that these transitions require strict regularization. We defined a simplified set of hyperparameters α_{flank}, α_{single} and α_{global} and (currently only roughly) searched for suitable values based on the quality of the produced alignments. These hyperparameters have a probabilistic foundation as parameters of Dirichlet priors over specific Bernoulli distributions that were defined to favor the probability $p = 1$ for particular, carefully defined events. That means the prior can be maximized by maximizing p, but this choice has to be balanced with the likelihood. For each possible choice of p and α the logarithmic prior densities are $(\alpha - 1)\ln p + (\alpha' - 1)\ln(1-p)$, where we set $\alpha' = 1$. The motivation behind this *ad hoc* choice was to keep the set of hyperparameters for the method simple while maintaining the probabilistic interpretation of the regularization term.

In particular, α_{flank} controls the pressure to align to the core model (rather than using the flanking states), i.e. increasing α_{flank} will result in longer insertions at the flanks and between repeated domain segments. The parameter α_{flank} regularizes the self-loop probabilities of all flanking states, as well as $P_0(L)$ and $P(R \mid E)$. Furthermore, we introduce α_{single} to penalize core model repeats favoring large values for the probability $1 - P(C \mid E) = P(R \mid E) + P(T \mid E)$. Lastly, α_{global} penalizes local alignments that use entry- and exit-transitions other than $S \to M_1$ and $M_l \to E$. The probabilities regularized by α_{global} were chosen such that all choices of starting and end points into the consensus $S \to M_i \to \cdots \to M_j \to E$ for $1 \le i \le j \le l$, $(i,j) \neq (1,l)$ are penalized uniformly. More precisely, we favor large probabilities $1 - P(M_i \mid S)P(E \mid M_j)$ for $1 \le i \le j \le l$, $(i,j) \neq (1,l)$. The values used for this paper are $\alpha_{flank} = 7000$, $\alpha_{single} = 1e9$ and $\alpha_{global} = 1e4$.

Initialization

First, we guess an initial model length l by taking the median of the sequence lengths and scaling it by a constant c. We found that $c = 0.8$ works well. It is easier to find a rough initial consensus if the number of match states is limited which forces the model to restrict itself to the more relevant parts of the sequences. The median is more robust against fragmentary sequences than the average.

The initialization of θ could in principle use prior knowledge about the protein family at hand. However, we are interested in *tabula rasa* training with an universal initial parameter set independent on the input sequences. We chose an *ad hoc* position independent initialization that reflects the prior distributions. Intuitively, we want the initial model to focus its probability mass on paths that use all match states. We do this by having larger probability for the initial match-match transitions. We took care to initialize the entry probabilities dependent of the model length such that $P(M_1 \mid S)$ is always roughly $\frac{1}{2}$. Moreover, we initialize the repeat transition $E \to C$ with a very small probability and for the flanking states L, R and C we initialize such that the self-loops are more likely than the exits.

Model surgery

After training, we might observe rarely used match states or overused insertion states. We can discard or expand those positions and adapt the model length which is known as *model surgery* [3].

Given a trained model, we discard match positions that are used by less than 50% of the sequences. Likewise, we expand positions where more than 50% of all sequences have an insertion by a number of new match states equal to the average insertion length. If a match position is discarded, all incident edges are removed and new edges with default initialization are carefully inserted to close the holes (there is a hole for each consecutive segment of discarded positions). If an insertion is expanded, edges at the position of interest that connect left and right model part are removed. Eventually, all edges incident to a new match state are default initialized. After each surgery iteration, the flanking states, θ_{init}, the kernel for the transition distribution of the end state E as well as the evolutionary times τ of the ancestral probability layer (for details see section 'Ancestral probabilities') are reset to default and the model is trained again. This is repeated at most 4 times which we found is a good compromise between speed and accuracy. Per default, we train 5 independent models and optimize them with model surgery. Eventually, we chose the model with parameters θ that maximizes $\frac{1}{m}(\mathcal{L}(\theta; S_1, \ldots, S_m) + \ln(\rho(\theta)))$ to decode the final alignment.

If the number of surgery iterations is > 1, we found it beneficial (both performance and accuracy wise) to restrict training in all but the last iterations to sequences with lengths above the q-th quantile while keeping a minimum of k sequences. Therefore, initial parameter updates are always on sequences that have roughly full-length. Short fragmentary sequences may disturb early training epochs. It is easier to incorporate them, if a rough consensus is established and the matter simplifies to fine-tuning the entry-, exit- and repeat-probabilities. We found that $q = 50\%$ and $k = 10.000$ work well. This is in line with other large scale MSA methods, where a common denominator is a strong preliminary focus on putative full-length sequences, i.e. sequences with lengths from the upper quantiles. For example, MAFFT-Sparsecore only considers sequences with lengths above the median for its core alignment and the regressive strategy favors the longest sequence as respresentatives of subtrees (i.e. longer sequences are aligned first).

Ancestral probabilities

We naturally assume the existence of a single whole-protein consensus sequence C that represents the sequence set we wish to align. Homologous sequences S_i may be closely or distantly related to C, i.e. we assume they have independent expected mutations per site with respect to the consensus. Model-wise we introduce evolutionary times τ_i to estimate the distance of S_i to C. The process is conventionally described by the General Time-Reversible Substitution Model parameterized by a 20×20 matrix Q of instantaneous substitution rates from one amino acid to any other [36, 37]. Like the scoring matrices used by traditional alignment algorithms, Q models prior biological knowledge on the relative expected frequencies of amino acid substitutions. From Q, the amino acid mutation probabilities after time τ given an initial amino acid can be derived as follows:

$$P(\tau) = \exp(\tau Q), \qquad (7)$$

where exp denotes the matrix exponential. The a-th row of this matrix, $P(\tau)_a$, corresponds to the expected amino acid distribution after time τ when starting with amino acid a. As the model is time-reversible, it is also the distribution of amino acids τ time units ago at a site where amino acid a is observed now.

We initialize τ with zeros and optimize it under the constraints $0 \leq \tau_S \leq 2.5$ where the maximal value of 2.5 corresponds to the PAM250 matrix and zero is the identity. The vector τ is learned jointly with the HMM parameters θ. Put differently, we learn the branch lengths of a star-like tree jointly with the sequence model. For each batch of sequences, the correct subset of τ is gathered. The ancestral probabilities with the final values τ are also used during Viterbi decoding of the alignment. More precisely, we replace all likelihoods $P(S_i \mid \theta)$ with $P(S_i \mid \theta, \tau_i)$.

The τ_i are related to sequence weights but they are learned from data and do not require a tree or any other pairwise sequence comparison. Assume that for some suitable distance metric one sequence S_i has a large total distance to all other sequences. In a sequence weighting scheme S_i would typically have a larger weight than sequences with many close relatives to account for the underrepresentation. Choosing a large τ_i can increase $P(S_i \mid \theta, \tau_i)$ by smearing S_i towards the consensus. But this increase is independent of all other sequences and involves no change of θ.

Technical background

We use TensorFlow [38] to automatically compute the gradients of ℓ with respect to θ and τ. We use the Adam optimizer [8] with a learning rate of 0.1 to minimize ℓ. Note that automatic differentiation allows low-effort changes to the HMM architecture and the prior. Moreover, the addition of any type of preliminary deep learning layer (e.g. ancestral probabilities) is possible. Using a machine learning back end provides access to GPU acceleration and other computational benefits out of the box. Our method does not strictly require a GPU, however, it is highly recommended to use one to train models beyond length 100. The training automatically scales to multiple GPUs by splitting the batches.

Data Description

We tested learnMSA on HomFam [11], BaliFam [39] and the ten largest Pfam [28] families. The former two are benchmark collections based on reference alignments from HOMSTRAD [40] and BAliBase [41] respectively. Each reference set is embedded into a large set of putative homologs gathered from Pfam. BaliFam has 2 variants where the references are embedded into 100 and 10.000 homologs respectively. Low sequence numbers were not our target of interest, but we included the small BaliFam variant specifically to test the up-scaling ability of our model. See Table 2 for further details. We did not modify, extend or reduce HomFam or BaliFam other than the embedding step as just described.

To test the ability of our method to align under high sequence length heterogeneity, we constructed a fragmentary variant of BaliFam10000 by following the procedure that was used to test UPP before [17]. We chose BaliFam10000, because the homologs had lengths comparable to the references whereas HomFam homologs in many cases appear to be not full-length. We constructed a high-fragmentation collection BaliFrag by randomly selecting 40% of the sequences per dataset in BaliFam10000. For each of these sequences, we sampled a fragment length from a normal distribution with mean equal to 33% of the mean length of the full-length sequences and a standard deviation of 15. We sampled uniformly from all valid starting positions of the fragment in the whole sequence.

Finally, we experimented with ten ultra-large datasets that were acquired from Pfam by selecting the largest families (based on the number of sequences in the full alignments) and downloading the respective UniProt datasets that were generated by searching the UniProtKB database using the Pfam family HMM. We also downloaded the corresponding seed alignments to use them as a reference. For the training datasets, we added the seed sequences to the UniProt datasets if not already present and removed all gaps. The families are: Zinc finger C2H2 type (PF00096), WD domain G-beta repeat (PF00400), ABC transporter (PF00005), Protein kinase domain (PF00069), Ankyrin repeats (PF12796), Major Facilitator Superfamily (PF07690), Leucine rich repeat (PF13855), Fibronectin type III domain (PF00041), Response regulator receiver domain (PF00072) and Immunoglobulin I-set domain (PF07679). All have known 3D structure. ABC transporter is the largest dataset with about 3.5 million sequences. See Table 3 for details.

Analysis

We compared learnMSA to the following aligners: Clustal Omega (Version 1.2.4), regressive T-Coffee (Version 13.45.0.4846264), MAGUS (git hash f9a3676 from 2022-01-21), UPP (Version 4.5.2) and MAFFT-Sparsecore (MAFFT

Table 2. Dataset properties

collection	number of families	number of sequences min	max	avg	sequence length min	max	avg
HomFam (refs.)	94	5	41	8	14	854	215
HomFam (combined)	94	93	93681	8007	12	854	148
BaliFam (refs.)	59	4	142	27	22	471	158
BaliFam100	59	104	242	127	20	764	161
BaliFam10000	36	10004	10142	10031	7	607	175
BaliFrag	36	10004	10142	10031	7	607	129

Table 3. Ultra-large dataset properties

family	no. sequences combined	seed	%id	sequence length min	max	avg
PF00005	3489586	55	26	18	683	146
PF07690	1861106	192	13	37	577	284
PF00096	1783511	159	41	12	34	23
PF00072	1767045	52	25	28	156	110
PF00400	1594257	1465	24	12	101	35
PF00069	1154714	38	21	24	511	227
PF12796	945198	184	24	27	153	78
PF13855	766271	62	28	26	73	57
PF00041	666310	98	20	27	139	81
PF07679	579519	48	21	25	149	83

Sequence identity is based on full alignment. Sequence lengths are given for the combined dataset.

Version 7.490). There is to the best knowledge of the authors no mature deep learning based tool for large multiple alignment of proteins available for comparison.

The command lines to align HomFam and BaliFam were (input/output and CPU arguments omitted):

```
MsaHmm.py
clustalo -t protein --outfmt=fa
mafft-sparsecore.rb
run_upp.py -M -1 -m amino
magus.py -t clustal --recursive false
t_coffee -reg -nseq 100 -tree mbed
         -method mafftginsi_msa
```

and for the ultra-large datasets (commands equal to the HomFam/BaliFam case omitted):

```
mafft --parttree
magus.py -t random --recurse True
         --recurseguidetree clustal
t_coffee -reg -nseq 1000 -tree parttree
         -method mafftfftnsi_msa
```

We run learnMSA as well as UPP on all datasets (including ultra-large) in default mode without manual parameter adjustments. We did not attempt to align the ultra-large files with Clustal Omega, because we already observed a severe drop in accuracy on sequences in the thousands. MAFFT-Sparsecore refused to align the ultra-large datasets. We used MAFFT with the parttree option instead. For MAGUS, we enabled recursion for the ultra-large datasets, set the guide tree for the highest recursion level to 'random' due to very long runtimes with other choices and used clustal trees for all other recursion levels. To use T-Coffee regressive on the ultra-large datasets, we increased the maximum number of sequences in the subalignments to 1000 in the hope that we could avoid very long MSAs due to concatenated independent gaps during the merging steps. For a speedup, we also run T-Coffee with parttree and MAFFT FFT-NS-i. All parameter changes in order to align the ultra-large datasets were done reactively after testing the slower and more accurate settings used for HomFam and BaliFam first.

Our method was run using 8 CPU cores, 100 GB of RAM and a NVIDIA GeForce RTX 3090 GPU for all datasets including the ultra-large ones. All other aligners did not utilize a GPU and were run using 8 CPU cores and 100 GB of RAM for HomFam and BaliFam and 16 cores and 500 GB of RAM for the ultra-large datasets. We chose all memory numbers as a safe upper limit and did no further experiments to evaluate tight requirements. We used a wall clock limit of 3 days for each individual ultra-large alignment.

Sum-of-pairs (SP) score and total column (TC) score were computed by comparing the subalignments induced by the reference sequences to a structure based alignment (in case of HomFam and BaliFam) or the Pfam seed alignment (in case of the ultra-large datasets). We used T-Coffee with the *aln_compare* option. The reference sequences are not known to the aligning method.

On the ultra-large datasets learnMSA is most accurate and fastest in almost all cases (see Table 4). All other methods except UPP required manual adjustment of the default parameters to get them to work. In the end, not all tested aligners were able to align all datasets indicating technical limitations of state-of-the-art tools. In addition to timeout and memory issues, we observed a tendency of the divide-and-conquer methods (T-Coffee, MAGUS) to construct MSAs with much larger column counts than the reference (see the expansion column in Table 4), sometimes to the extent that the output file was too large for further usage. This is most likely due to their merging of subalignments in which independent gaps are stacked rather than aligned. LearnMSAs alignments do not grow in length with increasing number of sequences. Figure 4 shows representatively that ultra-large MSAs computed by learnMSA tend to be tighter than those of comparable tools and do not suffer from underalignment. In the case of PF00096, learnMSA has no clear advantage, however, this family has relatively high sequence identity and very short sequences and is therefore easier to align than the others. Below 1 million sequences, learnMSA loses its runtime advantage and is about as fast as MAFFT and T-Coffee, but at the same time much more accurate.

Figure 2 shows the distribution of SP and TC scores for HomFam and BaliFam. We were able to match state-of-the-art performance on HomFam. If restricted to the 20 sequence sets with at least 10.000 sequences, the benefit of using pHMM based alignment increases. Note that the number of sequences in the HomFam collection varies significantly (see Table 2). Likewise, HMM matches state-of-the-art performance on BaliFam10000, but falls behind on BaliFam100.

LearnMSA aligned HomFam and BaliFam10000 in a total of 40 hours (sequential training of 5 independent models on the same machine). For the same, Clustal Omega took 3.5 hours, MAFFT-Sparsecore 24 hours, UPP 19 hours, T-Coffee regressive 9 hours and MAGUS 48 hours.

We also evaluated how increasing the number of homologs that are aligned together with the reference sequences affects alignment accuracy. To create a biologically realistic test set-

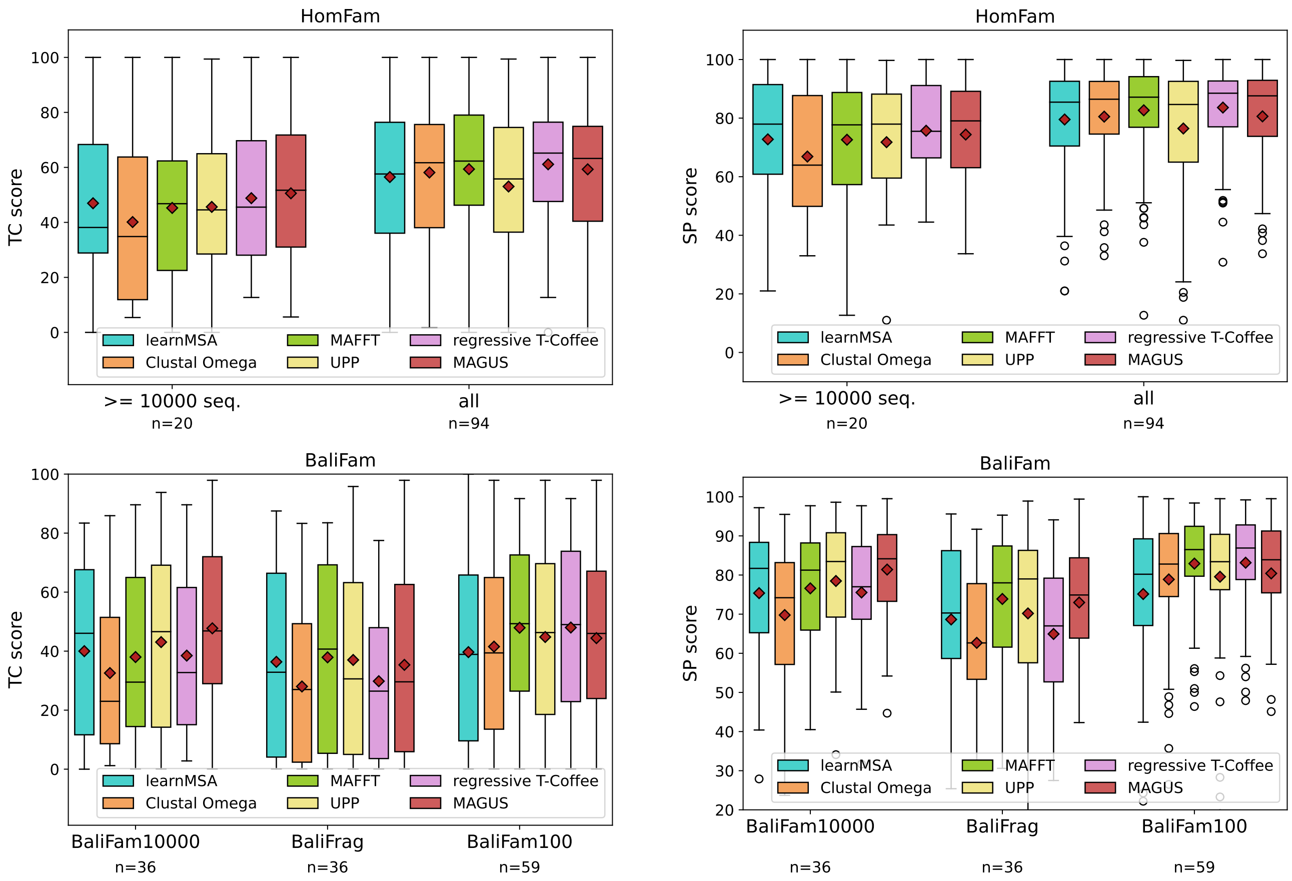

Figure 2. Total column (TC, left) and sum-of-pairs (SP, right) scores for the HomFam (top) and BaliFam (bottom) collections.

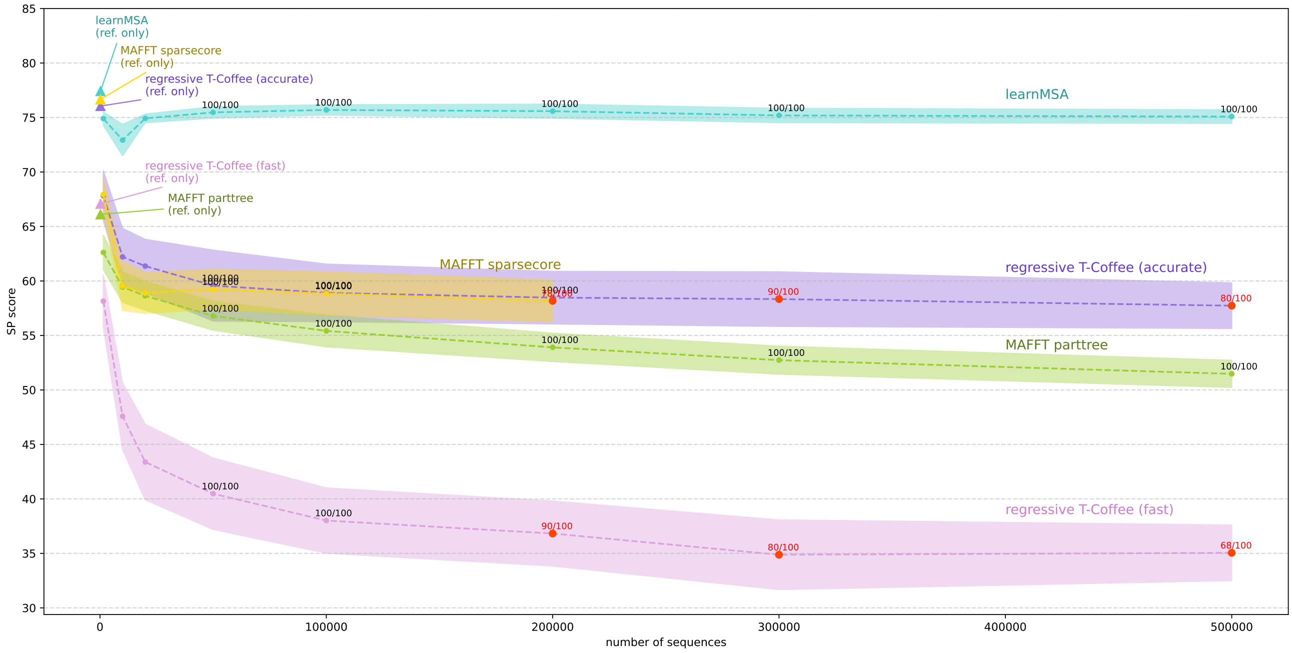

Figure 3. Alignment accuracy as a function of family size. For evaluation purposes, increasing numbers of further homologs are added to a static set of reference sequences. The data points are labeled with the fractions of alignment tasks that produced an usable MSA at all. Missing data points indicate that the aligning method failed for the entirety of the datasets. In case of a failed alignment (due to hardware constraints), we inserted the score of the largest successful alignment in the respective series of nested sets, in favor of the aligning method. Therefore, the plot shows the behavior of the accuracy of the remaining MSAs under the (obliging) assumption that the failed MSAs are in theory unaffected by an increase in sequence numbers. Such incomplete data points are colored red. The shaded area is the standard deviations over the 10 samples, averaged over the families.

Table 4. Results for the ultra-large datasets

family	method	SP	TC	hours	expansion
	learnMSA	**74.9**	**22.2**	**10.0**	**1.89**
	UPP	73.5	10.2	52.5	1.98
PF00005	MAFFT	error			
	MAGUS	timeout			
	regressive T-Coffee	error			
	learnMSA	**56.1**	0.0	**30.2**	**1.82**
	UPP	51.6	0.0	35.5	2.48
PF07690	MAFFT	error			
	MAGUS	timeout			
	regressive T-Coffee	error			
	learnMSA	92.9	6.5	0.9	**1.16**
	UPP	86.3	0.0	1.7	2.23
PF00096	MAFFT	84.1	**16.1**	**0.3**	2.74
	MAGUS	**94.8**	3.2	3.6	4.68
	regressive T-Coffee	69.9	0.0	0.9	6.55
	learnMSA	**92.4**	**39.2**	**2.9**	**1.1**
	UPP	91.4	34.6	6.7	1.32
PF00072	MAFFT	64.9	4.6	7.6	3.69
	MAGUS	85.8	33.1	24.8	2.41
	regressive T-Coffee	output too large			
	learnMSA	**18.0**	0.0	**1.1**	**1.29**
	UPP	3.6	0.0	2.0	2.62
PF00400	MAFFT	0.0	0.0	2.3	7.71
	MAGUS	6.9	0.0	12.6	17.32
	regressive T-Coffee	0.0	0.0	2.0	51.28
	learnMSA	**83.4**	**24.9**	**11.3**	**1.37**
	UPP	83.3	20.2	19.5	1.6
PF00069	MAFFT	54.9	5.4	53.0	3.52
	MAGUS	65.4	18.1	29.1	4.77
	regressive T-Coffee	error			
	learnMSA	**72.4**	0.0	**1.3**	**0.85**
	UPP	40.8	0.0	4.3	3.18
PF12796	MAFFT	40.4	**0.4**	7.5	6.36
	MAGUS	58.9	0.0	67.2	5.62
	regressive T-Coffee	output too large			
	learnMSA	**94.7**	26.2	**0.8**	**1.05**
	UPP	91.0	21.5	2.5	1.71
PF13855	MAFFT	80.6	3.1	1.2	3.05
	MAGUS	**94.7**	**38.5**	54.1	1.47
	regressive T-Coffee	49.2	0.0	**0.8**	7.21
	learnMSA	**79.1**	16.5	1.0	**1.34**
	UPP	74.9	**22.0**	2.3	2.18
PF00041	MAFFT	43.2	0.0	2.0	7.83
	MAGUS	72.6	10.1	53.8	6.4
	regressive T-Coffee	37.0	0.0	**0.8**	15.16
	learnMSA	**94.1**	**50.0**	0.9	**1.11**
	UPP	88.7	46.0	2.9	1.43
PF07679	MAFFT	68.1	13.0	1.1	3.36
	MAGUS	84.0	42.0	4.3	2.12
	regressive T-Coffee	44.2	2.0	**0.6**	8.55

Expansion denotes the ratio of the length of the predicted alignment (induced by the reference sequences) to the reference alignment length. Values greater than 1 indicate underalignment i.e. the estimated alignment is longer than the reference. Timeout: The alignment could not be completed by the method within a wall clock limit of 3 days. Error: The alignment failed with an error (either out of memory or another unknown reason). Output too large: The alignment was successful, but the output file was impractically large to be properly post-processed (for example PF12796: T-Coffee 445GB, learnMSA 1.2GB). For each cell and column, the best value is in bold face.

ting, we took the 10 Pfam datasets from Table 3 and aligned the combination of the respective seed sequences (called references in the following) with random subsets of the remaining homologs. We started by aligning only the references. Note that the reference set sizes vary between 38 and 1465 (Table 3). Homologs were drawn randomly without replacement from the UniProtKB datasets to fill up the aligned datasets to monotonically increasing sizes, such that the resulting sets are nested (in a series of MSAs, homologs are only added, never removed). We repeated this serial sampling procedure 10 times and averaged the results over equal sized alignments. We compared learnMSA with T-Coffee regressive and MAFFT using the commands above from previous experiments, both the accurate and fast variants.

As seen in Figure 3, the accurate variant of T-Coffee regressive, MAFFT-Sparsescore and learnMSA are similar in SP score when only aligning the references. Further, all alignment methods lose accuracy after adding homologs at all. However, the asymptotic accuracy of learnMSA is barely affected by the number of added homologs, whereas we observe clearly decreasing trends for the other methods. The relative performance of the methods is dataset dependent and indicates that learnMSA has advantages for the global alignment of protein families. Starting at 200.000 sequences, we observed that regressive T-Coffee and MAFFT sparsecore failed for some MSA tasks (we allowed 200 GB of RAM per MSA). The only methods able to align all datasets were learnMSA and MAFFT with the partree option. For our evaluation, we decided to replace each failed MSA with the largest successful alignment in the respective series of nested sets, assuming, in favor of the aligning method, that the failed MSA is in theory unaffected by an increase in sequence numbers. Despite that, as seen in Figure 3, typically a further increase in the number of homologs still leads to a decrease in accuracy of the established algorithmic aligners.

For the high-fragmentation collection BaliFrag, learnMSA can compete with MAFFT-Sparsecore, UPP and MAGUS (Figure 2). All rely on robust ways to exclude putative fragmentary sequences in early alignment stages by restricting initial backbone alignments to sequences from the upper quantiles [13, 17, 15]. Clustal Omega and T-Coffee regressive fall behind in this benchmark. This analysis confirms that learnMSA can accurately adapt to fragmentary sequences by first training a pHMM on sequences that are deemed full-length and fitting to the complete sequence set thereafter. Partial domain hits correctly use the entry- and exit-transitions as seen in Figure 5. The difference of learnMSA to the competing methods is that we do not restrict the initial stages to a constant-sized subset of the sequences and that the final alignment is, in principle, able to correct incorrect decisions from earlier iterations. A suitable number of full-length examples is required to find a correct initial model length and to build a consensus. However, UPP teaches us that it is easy to add fragmentary sequences with pHMMs once a full-length consensus is established [17].

Discussion

We have proposed learnMSA, a novel unsupervised learning approach for the alignment of large protein families. In contrast to state-of-the-art aligners, learnMSA does not require a tree, which eliminates a crucial performance bottleneck and makes learnMSA asymptotically fast – linear in the number of sequences. It is interesting to see that state-of-the-art performance on large sequence numbers can be reached without a tree by uniform batch sampling. Our method does not rely on progressive, regressive or divide-and-conquer heuristics. We showed empirically, that learnMSA, when aligning millions of sequences, is both more accurate and faster (even though the measured time was for 5 independent, sequentially trained models). Moreover, when aligning Pfam families, additional homologs decrease the accuracy of traditional, heuristic methods (if they are feasible for large sequence numbers at all), whereas learnMSA is more robust. Whether this statement also applies to established benchmarks like HomFam remains an open question that can be answered if more homologs are gathered for these datasets in the future. A similar scaling experiment, which was done for T-Coffee regressive [10] based on HomFam, suffers from limited data coverage for large sequence numbers, i.e. the number of available families decreases when the MSA depth increases. This is not the case in our study as

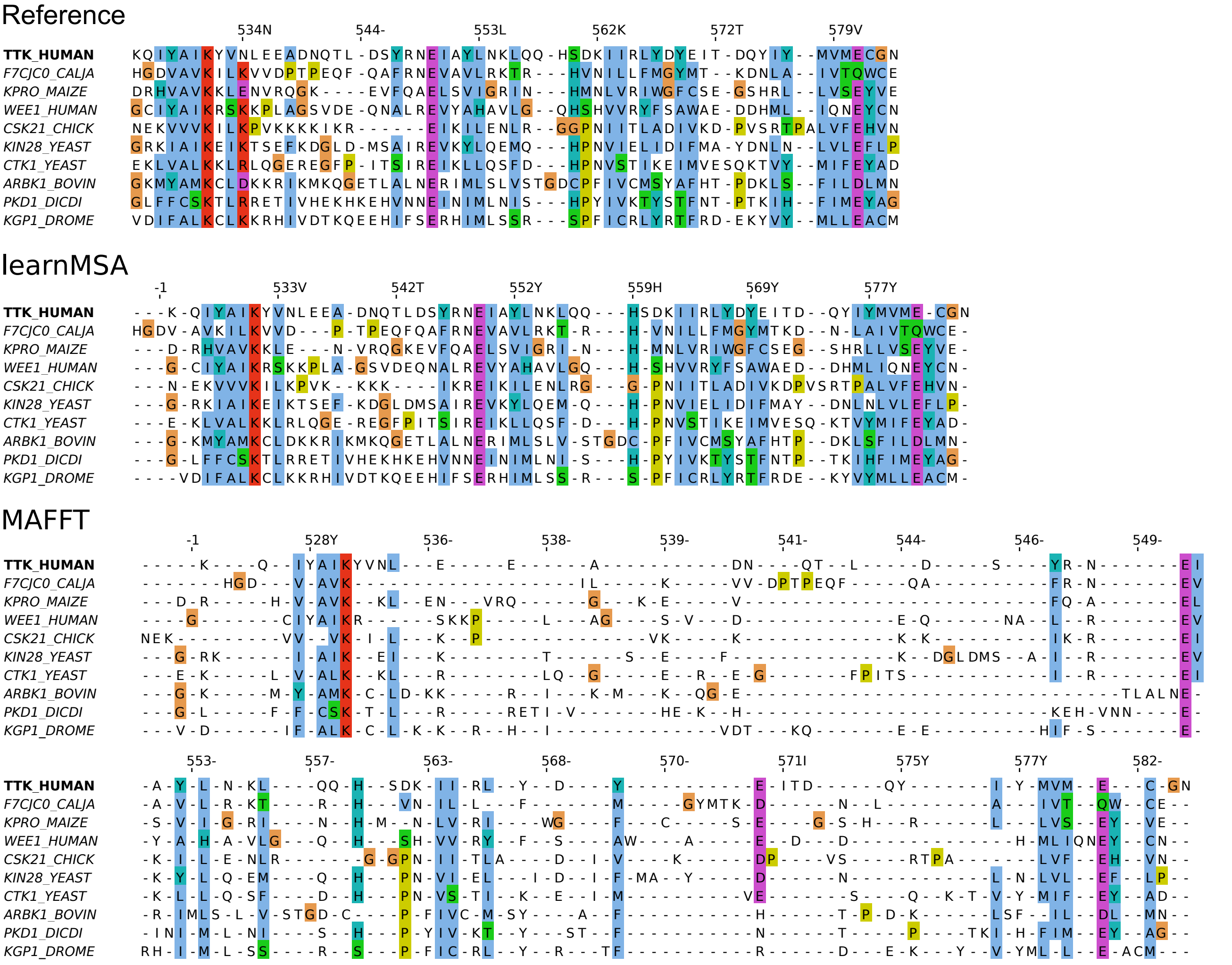

Figure 4. Vertical MSA slices for the ultra-large family PF00069 with more than a million sequences. The 10 most informative sequences (i.e. the most dissimilar ones based on the reference MSA) were extracted using T-Coffee. We took a random vertical slice ranging from column 25 to 90 in the reference MSA and computed vertical slices for the predicted MSAs as induced by the sequence fragments. We used Jalview 2.11.2.2 with clustalx coloring for visualization. For better comparability, TTK_HUMAN was selected as reference sequence.

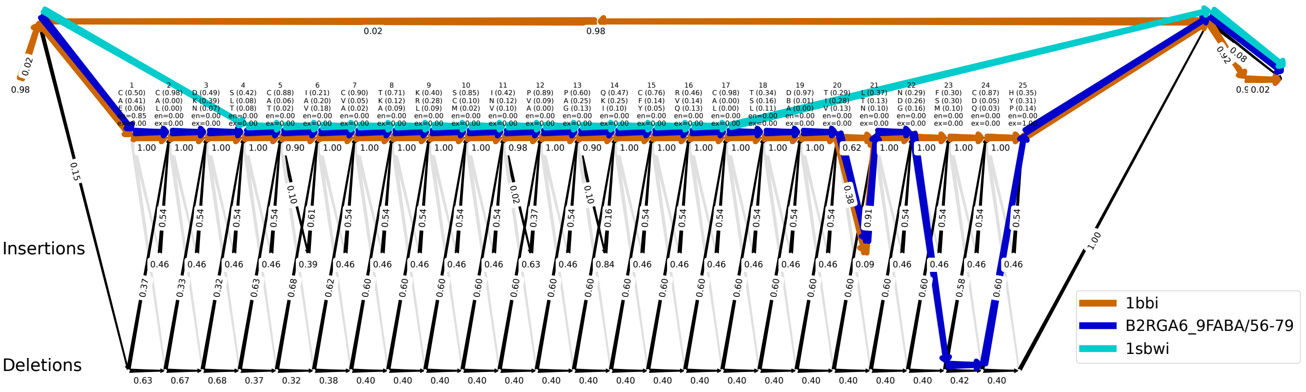

Figure 5. A learned pHMM for the Bowman-Birk serine protease inhibitor family in the HomFam collection with Viterbi paths for three different sequences: A single domain hit (blue), a multihit (brown) and a partial hit (cyan). Numbers on edges are transition probabilities, numbers on nodes are self-loop probabilities. For each match states, the top three amino acids and their probabilities are printed, along with the probability of entering and exiting at the respective match.

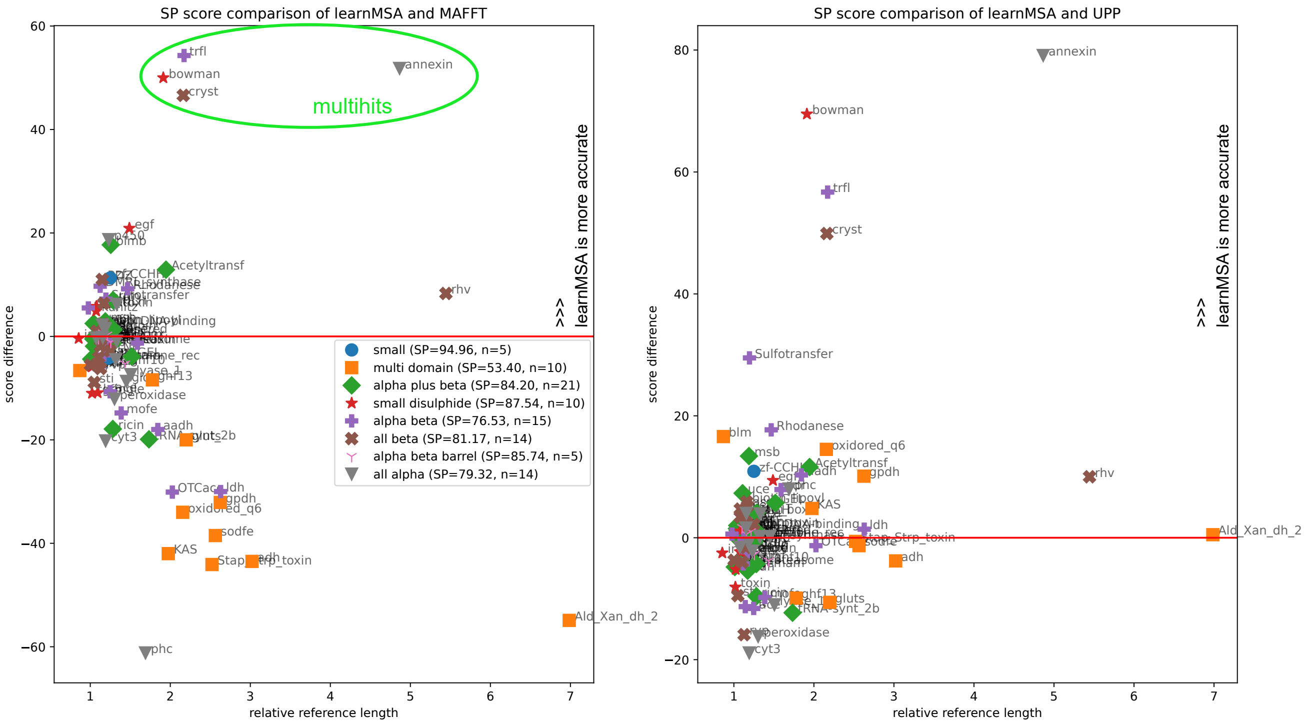

Figure 6. A detailed comparison of the performance of learnMSA relative to MAFFT-Sparsecore (left) and UPP (right) for all 94 HomFam families grouped by secondary structure. Score difference is defined as $SP(learnMSA) - SP(other)$. Relative reference length is defined as the ratio of the average reference length and the average length of the combined dataset including the homologs. For example, 'Ald_Xan_dh_2' references are on average about 7 times as long as the respective homologs. The legend contains the average SP score of learnMSA per structure group.

enough homologs were available from the UniProtKB datasets.

LearnMSA generalizes and automatizes earlier pHMM training approaches for protein families. It does this by taking HMMER's 'Plan7' model, but avoids the manual adjustment of the 'alignment mode' (local versus glocal or unihit versus multihit). Instead, the extra states and transitions (orange in Figure 1 A) are optimized jointly with the core model starting with a *tabula rasa* configuration which greatly reduces the required hand-holding. This is also beneficial, if a suitable alignment mode for a dataset is unknown. LearnMSA is designed in a way that minimizes the assumptions a user has to make. Note that for all tested datasets, including dramatically varying sequence numbers and levels of fragmentation, we used learnMSAs default configuration of hyperparameters. It should be pointed out, that learnMSAs is particularly accurate compared to other methods when aligning families that contain multihits. This is clearly visible in Figure 6, for example in the cases of Beta gamma crystallin ('cryst', PF00030), Bowman-Birk protease inhibitor ('bowman', PF00228) or Annexin ('annexin', PF00191).

On HomFam and BaliFam we match state-of-the-art performance but observe reduced relative accuracy for low sequence numbers. This indicates that there is a lower limit on the sequence numbers below which learnMSAs performance decreases relatively to other methods, but this is not surprising for a statistical learning approach and can currently be solved by falling back to a traditional aligner. There is a slight disadvantage of HMM in average scores for HomFam over all 94 datasets compared to only the largest 20. HomFam contains datasets with a few as 93 sequences. Further evaluation revealed that the disadvantage is not fully explained by low sequence numbers alone, however. Instead, we observed problems if the reference sequences are significantly longer than the homologs (for instance rhv references are on average five times as long as the homologs). Figure 6 (left) indicates a negative correlation between relative reference length with respect to homologs and score difference. The low-score cases frequently map to 'multi domain' secondary structures. In those cases the references are full-length proteins and the homologs pruned to a specific domain (i.e. information is cut away). This effect is present for all comparison tools except UPP which is shown in Figure 6 on the right. For statistical learning the choice of homologs in HomFam constitutes a problem. The number of reference sequences is very low (8 on average for HomFam) and they can contain information that the homologs miss, which means that potentially important motifs are underrepresented in the dataset. In such situations it is both hard to guess a suitable initial model length and train a full-length model from scratch. Moreover, this reveals a potential weak spot of the HomFam collection: A method that aligns the longest sequences in a dataset first, will most likely catch the references early. The score, which is estimated on the references only, might therefore overestimate the true score on the complete dataset.

Note that in principle, learnMSA could also align DNA/RNA sequences, but this feature is not implemented yet. Machine learning methods can likely play out their advantages more for proteins due to the relative complexity of parameter space and priors. Further, learnMSA is currently best suited for short or medium length sequences.

Conclusion

Our proposed approach constitutes a probabilistically grounded framework for large MSA that has potential for further improvements in several directions. Further development might be straightforward because of the extensible nature of our method.

A natural extension of the work presented here are ensembles of pHMMs. They are used in UPP where a subset of the sequences is aligned and subsequently represented by an ensem-

ble. Recently, MAGUS combined with an HMM ensemble has shown improved accuracy as well [42]. On the HomFam collection, UPPs performance decreased slightly when replacing the ensemble with a single HMM [17]. The latter is related to our approach with the difference that for learnMSA, the HMM parameters depend on all input sequences instead of a randomly selected backbone set. This might explain why learnMSA aligns HomFam slightly more accurately than UPP, as seen in Figure 2, even though learnMSA does not currently use an ensemble.

When benchmarking learnMSA, we observed decreasing relative performance when reducing the number of sequences to align. The behavior of state-of-the-art tools is usually complementary: They are more accurate for lower sequence numbers. Moreover, Figure 6 shows that the relative performance of learnMSA greatly depends on the particular (reference) dataset. This suggests the idea of a combined approach to multiple sequence alignment, where a prior (e.g. the number of sequences) or posterior (e.g. the likelihood) criterion is used to decide between the MSA of either learnMSA or of an established heuristic aligner.

In contrast to traditional learning algorithms for HMMs, gradient-based learning can, in principle, be a module of a larger machine learning model that is trained end-to-end. By design, learnMSA can incorporate any type of sequence context encoded into the HMM alphabet. For instance, single-sequence secondary structure predictions can be incorporated. Secondary structure is more conserved than primary sequence and this approach has been shown to increase accuracy in the presence of low sequence identity [43]. There are many kinds of interactions in proteins that are not easily modeled by our current approach, for example pairwise correlations between amino acid distributions in positions that are widely separated in the primary sequence but close in the three-dimensional structure. The field of protein language modeling where parameter-rich sequence models are learned semi-supervised [44, 45] based on Attention [46, 47] or LSTMs [48] is also compatible and complementary to our approach. Currently, we use very limited prior knowledge about proteins in the form of parameters as we simply one-hot encode amino acids and only use a rate matrix to compute ancestral probabilities. Using instead semantically rich [44] residual-level embedding vectors from pre-trained language models may benefit the predictions.

Availability of source code and requirements

- Project name: learnMSA
- Project home page: https://github.com/Gaius-Augustus/learnMSA
- Operating system(s): Platform independent
- Programming language: Python3
- Other requirements: Python packages tensorflow, optional for visualization: networkx, logomaker
- License: MIT
- RRID: SCR_022572
- biotoolsID: learnMSA

Availability of supporting data and materials

The datasets supporting the results of this article are available in the repository https://github.com/felbecker/MSA-HMM-Analysis. An archival copy of code and data is also available via the GigaScience database GigaDB [49].

List of abbreviations

(p)HMM: (profile) hidden Markov model; MSA: multiple sequence alignment

Competing Interests

The authors declare that they have no competing interests.

Author's Contributions

F.B. designed and implemented learnMSA, prepared the data, ran all software and wrote the manuscript. M.S. conceived the idea and designed and implemented an initial version of a recurrent machine learning layer for HMMs and provided prototype code for the usage of ancestral probabilities. All authors approved the final manuscript.

References

1. Eddy SR. Accelerated profile HMM searches. PLoS computational biology 2011;7(10):e1002195.
2. Mistry J, Finn RD, Eddy SR, Bateman A, Punta M. Challenges in homology search: HMMER3 and convergent evolution of coiled-coil regions. Nucleic acids research 2013;41(12):e121–e121.
3. Krogh A, Brown M, Mian IS, Sjölander K, Haussler D. Hidden Markov models in computational biology: Applications to protein modeling. Journal of molecular biology 1994;235(5):1501–1531.
4. Eddy SR, et al. Multiple alignment using hidden Markov models. In: Ismb, vol. 3; 1995. p. 114–120.
5. Baldi P, Chauvin Y, Hunkapiller T, McClure M. Hidden Markov models in molecular biology: new algorithms and applications. Advances in Neural Information Processing Systems 1992;5.
6. Rasmussen TK, Krink T. Improved Hidden Markov Model training for multiple sequence alignment by a particle swarm optimization—evolutionary algorithm hybrid. Biosystems 2003;72(1-2):5–17.
7. Sun J, Wu X, Fang W, Ding Y, Long H, Xu W. Multiple sequence alignment using the Hidden Markov Model trained by an improved quantum-behaved particle swarm optimization. Information Sciences 2012;182(1):93–114.
8. Kingma DP, Ba J. Adam: A method for stochastic optimization. arXiv preprint arXiv:14126980 2014;.
9. Baydin AG, Pearlmutter BA, Radul AA, Siskind JM. Automatic differentiation in machine learning: a survey. Journal of Marchine Learning Research 2018;18:1–43.
10. Garriga E, Di Tommaso P, Magis C, Erb I, Mansouri L, Baltzis A, et al. Large multiple sequence alignments with a root-to-leaf regressive method. Nature biotechnology 2019;37(12):1466–1470.
11. Sievers F, Wilm A, Dineen D, Gibson TJ, Karplus K, Li W, et al. Fast, scalable generation of high-quality protein multiple sequence alignments using Clustal Omega. Molecular systems biology 2011;7(1):539.
12. Katoh K, Standley DM. MAFFT multiple sequence alignment software version 7: improvements in performance and usability. Molecular biology and evolution 2013;30(4):772–780.
13. Yamada KD, Tomii K, Katoh K. Application of the MAFFT sequence alignment program to large data—reexamination of the usefulness of chained guide trees. Bioinformatics 2016;32(21):3246–3251.
14. Mirarab S, Nguyen N, Guo S, Wang LS, Kim J, Warnow T.

PASTA: ultra-large multiple sequence alignment for nucleotide and amino-acid sequences. Journal of Computational Biology 2015;22(5):377–386.
15. Smirnov V, Warnow T. MAGUS: multiple sequence alignment using graph clustering. Bioinformatics 2021;37(12):1666–1672.
16. Smirnov V. Recursive MAGUS: scalable and accurate multiple sequence alignment. PLoS computational biology 2021;17(10):e1008950.
17. Nam-phuong DN, Mirarab S, Kumar K, Warnow T. Ultra-large alignments using phylogeny-aware profiles. Genome biology 2015;16(1):1–15.
18. Katoh K, Toh H. PartTree: an algorithm to build an approximate tree from a large number of unaligned sequences. Bioinformatics 2007;23(3):372–374.
19. Price MN, Dehal PS, Arkin AP. FastTree 2 – approximately maximum-likelihood trees for large alignments. PloS one 2010;5(3):e9490.
20. Jafari R, Javidi MM, Kuchaki Rafsanjani M. Using deep reinforcement learning approach for solving the multiple sequence alignment problem. SN Applied Sciences 2019;1(6):1–12.
21. Kuang M, Liu Y, Gao L. DLPAlign: A Deep Learning based Progressive Alignment Method for Multiple Protein Sequences. In: CSBio'20: Proceedings of the Eleventh International Conference on Computational Systems-Biology and Bioinformatics; 2020. p. 83–92.
22. Song YJ, Ji DJ, Seo H, Han GB, Cho DH. Pairwise heuristic sequence alignment algorithm based on deep reinforcement learning. IEEE open journal of engineering in medicine and biology 2021;2:36–43.
23. Llinares-López F, Berthet Q, Blondel M, Teboul O, Vert JP. Deep embedding and alignment of protein sequences. bioRxiv 2021;.
24. Jumper J, Evans R, Pritzel A, Green T, Figurnov M, Ronneberger O, et al. Highly accurate protein structure prediction with AlphaFold. Nature 2021;596(7873):583–589.
25. Mirabello C, Wallner B. RAWMSA: End-to-end deep learning using raw multiple sequence alignments. PloS one 2019;14(8):e0220182.
26. Fukuda H, Tomii K. DeepECA: an end-to-end learning framework for protein contact prediction from a multiple sequence alignment. BMC bioinformatics 2020;21(1):1–15.
27. Ju F, Zhu J, Shao B, Kong L, Liu TY, Zheng WM, et al. CopulaNet: Learning residue co-evolution directly from multiple sequence alignment for protein structure prediction. Nature communications 2021;12(1):1–9.
28. Mistry J, Chuguransky S, Williams L, Qureshi M, Salazar GA, Sonnhammer EL, et al. Pfam: The protein families database in 2021. Nucleic acids research 2021;49(D1):D412–D419.
29. Eddy SR. Profile hidden Markov models. Bioinformatics (Oxford, England) 1998;14(9):755–763.
30. Eddy SR. A probabilistic model of local sequence alignment that simplifies statistical significance estimation. PLoS computational biology 2008;4(5):e1000069.
31. Rabiner L, Juang B. An introduction to hidden Markov models. ieee assp magazine 1986;3(1):4–16.
32. Durbin R, Eddy SR, Krogh A, Mitchison G. Biological sequence analysis: probabilistic models of proteins and nucleic acids. Cambridge university press; 1998.
33. Van der Auwera S, Bulla I, Ziller M, Pohlmann A, Harder T, Stanke M. ClassyFlu: classification of influenza A viruses with Discriminatively trained profile-HMMs. PLoS One 2014;9(1):e84558.
34. Brown M, Hughey R, Krogh A, Mian IS, Sjölander K, Haussler D. Using Dirichlet mixture priors to derive hidden Markov models for protein families. In: Ismb, vol. 1; 1993. p. 47–55.
35. Sjölander K, Karplus K, Brown M, Hughey R, Krogh A, Mian IS, et al. Dirichlet mixtures: a method for improved detection of weak but significant protein sequence homology. Bioinformatics 1996;12(4):327–345.
36. Dayhoff MO, Eck R, Park C. A model of evolutionary change in proteins. Atlas of protein sequence and structure 1972;5(88-99):88–99.
37. Le SQ, Gascuel O. An improved general amino acid replacement matrix. Molecular biology and evolution 2008;25(7):1307–1320.
38. Abadi M, Barham P, Chen J, Chen Z, Davis A, Dean J, et al. TensorFlow: A System for Large-Scale Machine Learning. In: 12th USENIX symposium on operating systems design and implementation (OSDI 16); 2016. p. 265–283.
39. Edgar RC. MUSCLE v5 enables improved estimates of phylogenetic tree confidence by ensemble bootstrapping. bioRxiv 2021;.
40. Stebbings LA, Mizuguchi K. HOMSTRAD: recent developments of the homologous protein structure alignment database. Nucleic acids research 2004;32(suppl_1):D203–D207.
41. Thompson JD, Koehl P, Ripp R, Poch O. BAliBASE 3.0: latest developments of the multiple sequence alignment benchmark. Proteins: Structure, Function, and Bioinformatics 2005;61(1):127–136.
42. Shen C, Zaharias P, Warnow T. MAGUS+ eHMMs: improved multiple sequence alignment accuracy for fragmentary sequences. Bioinformatics 2022;38(4):918–924.
43. Wright ES. DECIPHER: harnessing local sequence context to improve protein multiple sequence alignment. BMC bioinformatics 2015;16(1):1–14.
44. Alley EC, Khimulya G, Biswas S, AlQuraishi M, Church GM. Unified rational protein engineering with sequence-based deep representation learning. Nature methods 2019;16(12):1315–1322.
45. Rao R, Bhattacharya N, Thomas N, Duan Y, Chen X, Canny J, et al. Evaluating protein transfer learning with TAPE. Advances in neural information processing systems 2019;32:9689.
46. Vaswani A, Shazeer N, Parmar N, Uszkoreit J, Jones L, Gomez AN, et al. Attention is all you need. Advances in neural information processing systems 2017;30.
47. Devlin J, Chang MW, Lee K, Toutanova K. Bert: Pre-training of deep bidirectional transformers for language understanding. arXiv preprint arXiv:181004805 2018;.
48. Hochreiter S, Schmidhuber J. Long short-term memory. Neural computation 1997;9(8):1735–1780.
49. Becker F, Stanke M. Supporting data for "learnMSA: Learning and Aligning Large Protein Families". GigaScience Database; 2022, http://dx.doi.org/10.5524/102320.
