# Supplementary material for: learnMSA: learning and aligning large protein families
Source: Gigascience. 2022 Nov 18;11:giac104. doi: 10.1093/gigascience/giac104 (PMC9673500; doi:10.1093/gigascience/giac104)
Supplement: giac104_Response_to_Reviewer_Comments_Original_Submission [file giac104_response_to_reviewer_comments_original_submission.pdf]

Author's Response To Reviewer Comments

Close

Dear Hans,

We thank the reviewers for the extensive and constructive criticism. We believe we have fully addressed the reviewers and your concerns and issues and included two versions of the manuscript, the second with changes marked in red. Importantly, our tool is now easily installable via both PyPI/pip and Bioconda. Moreover, we have performed additional experiments to support one of our arguments, where Reviewer #2 was “not convinced” and “suggested a rewording” only. We decided to elaborate more on this concern, added a new Figure 3 and believe these additional results strengthen the manuscript. Further, we registered learnMSA with bio.tools and scicrunch.org.

We address the reviewers’ comments point by point below.

Reviewer #1: The article describes an original method, learnMSA, for construction of large multiple sequence alignments, that uses a recurrent neural network approach to learn profile Hidden Markov models of protein sequence families. The method is evaluated, and compared to state of the art methods, on existing benchmarks containing very large test sets, some with more than a million sequences. The methods and evaluation experiments are clearly described and the results indicate that learnMSA is competitive in terms of alignment accuracy and calculation time.

Major criticisms:
1. Other recent work using deep learning approaches to construct multiple sequence alignments should be discussed, and if possible included in the comparisons. For example, Zhang et al. DeepMSA: constructing deep multiple sequence alignment to improve contact prediction and fold-recognition for distant-homology proteins. Bioinformatics. 2020; Kuang et al. DLPAlign: A Deep Learning based Progressive Alignment Method for Multiple Protein Sequences CSBio2020: CSBio '20: Proceedings of the Eleventh International Conference on Computational Systems-Biology and Bioinformatics; Jafari et al. Using deep reinforcement learning approach for solving the multiple sequence alignment problem. SN Applied Sciences volume 1, Article number: 592 (2019)

Authors: We thank the reviewer and have added a deep learning paragraph to the introduction with 8 additional references. However, the first reference that Reviewer #1 listed (DeepMSA) does not present a machine learning method although its name may suggest so. We could not include any deep learning based tools in the comparison as there are no such mature tools, only prototypes or proof of concepts.

Reviewer #1: I tried to install and run the software (using Tensorflow 2.5.0), but it failed with the following error message: "NotImplementedError: Cannot convert a symbolic Tensor (msa_hmm_layer/strided_slice_47:0) to a numpy array. This error may indicate that you're trying to pass a Tensor to a NumPy call, which is not supported".

Authors: We apologize for the inconveniences with the manual installation of learnMSA and its dependencies. We could reproduce this error and found out it was due to a version conflict with another package which TensorFlow depends on. To avoid such problems in the future, we now built and deposited learnMSA as a package at the Python Package Index (PyPi), which can be installed from the prompt by typing ‘pip install learnMSA’. Alternatively, our tool can be installed using Bioconda preferably in a clean conda environment with ‘conda create -n learnMSA learnMSA’ assuming that the Bioconda channel is set up. Please see https://github.com/Gaius-Augustus/learnMSA for detailed installation instructions.

Reviewer #1: More minor comments:
1. The method is demonstrated using protein sequences. Is it also suitable for the alignment of DNA/RNA sequences?

Authors: In principle, learnMSA could also align DNA/RNAsequences, but this feature is not implemented yet. Machine learning methods like profile HMMs can likely play out their advantages for proteins due to the relative complexity of parameter space and priors. We have added this comment to the Discussion in the manuscript.

Reviewer #2: The authors present a practically applicable implementation of a hitherto unexplored approach for the multiple sequence alignment problem that was first described in the 1990's by Eddy, Krogh, Sjolander, et al.. learnMSA takes advantage of tensorflow to perform 'statistical alignment' by iterations of steepest descent and algorithmic pruning to identify a single optimal profile hidden markov model for a set of sequences. The pHMM model advances Eddy's Plan7 architecture in its support for both ancestral state and repeat regions, and the accompanying code provides mechanisms for HMM model visualisation in addition to emission of the multiple alignment of the given sequences induced by the final model.

Authors: Thanks

Reviewer #2: Code. The authors provide a repository containing a python module (with tests), a python script for command line execution, and a jupyter notebook demonstrating the methodology and results visualisation. Whilst documentation is sparse, the code performs as described. I look forward to the package being made available via pip and ultimately bioconda. I also look forward to enhancements made by the authors and the future learnMSA community that enable users to make use of the additional data embodied by the learned pHMM.

Authors: We have now made learnMSA easily available as a command line tool via both PyPI/pip and Bioconda. Please see https://github.com/Gaius-Augustus/learnMSA for detailed installation instructions. The tool will be in active development beyond the paper release.

Reviewer #2: Manuscript. Overall, the manuscript presents a clear account of the theoretical approach and practical implementation. Clarity could be improved in some areas, and suggestions are made below. The authors also devised challenging benchmarks in order to evaluate their method, which demonstrated both its strengths and potential weaknesses. Whilst the results are convincing, they necessarily rely on MSA statistics that are difficult to interpret, but this should not be a barrier to publication. Ideally, a more robust analysis could be performed with gold standard data such as structures, perhaps by adapting established MSA benchmarking tools such as OxBench.

Authors: Thank you for suggesting additional benchmarks to challenge learnMSA. The reference alignments from HomStrad and BaliBase we chose for evaluation are both already structure-based. To extrapolate accuracy on large sequence numbers, manually selected homologs were added (by others, in other publications) to create the extended benchmarks HomFam and BaliFam which we used. OxBench's alignments are again too shallow for themselves (2 to 122 sequences) to be a proper benchmark for our focus on large sequence families. Manually adding homologs to OxBench would require constructing a new benchmark altogether which we consider out of the scope of our manuscript. We argue that two different benchmarks with structure based reference alignments in combination with the large datasets from Pfam are already quite suitable to alleviate dataset-specific biases in the evaluation.

Reviewer #2: Below I note a number of questions for the authors, followed by suggested revisions, and finally a handful of grammar/typo fixes.

Q1. are the disadvantages regarding domain repeats (in Viterbi decoding) addressable ?

Authors: Indeed, this is a good point to raise. Currently, our approach does not make explicit use of differences between the copies when a protein contains a domain multiple times. If multiple copies occur, possibly one could do a higher-level alignment, in which the characters are domain occurrences. Some suitable score for a pair of domain occurrences has to be defined for that. We consider this an idea for future improvements.

Reviewer #2: Q2. the model surgery employs a 50% threshold for discard of underpopulated match states or over-represented insertions - are there situations where this could cycle ? If so, can such pathologies be detected in the reported statistics for the model ? Could these heuristics also cause the

problems when aligning sequences of greatly differing lengths ?

Authors: In a theoretical worst case scenario this could cycle, but only under very unlucky conditions. Currently, this can be detected manually from the default output of learnMSA which includes information about which positions were extended or discarded after each iteration. Re-running learnMSA with larger thresholds (e.g. 60%) should then fix the problems. For the software release accompanying the paper, we limit the number of surgery iterations to at most 4, such that a cycling surgery does probably little harm at all.
Concerning greatly differing sequence lengths, assuming that in a hypothetical scenario when about 50% of the sequences are full-length and the others are short fragments mapping to roughly the same segment of the protein, learnMSA has to decide between a long model, where the fragments use the entry/exit-distribution or a short model, where the flanks of the full length sequences are insertions. The long model can accommodate the fragments rather cheaply, whereas in the short model the flanks would be more expensive because they would be modeled as emission from a background distribution. LearnMSA could indeed cycle in this specific case, but we generally do multiple independent training runs and if one of the resulting models is by chance the long model with higher score, it will be selected automatically.

Reviewer #2: Q3. The command line tool only supports output of the final MSA - is there utility in a) reporting also the pHMM for the MSA and b) the ancestral probabilities ?

Authors: We have implemented a command line option to output the learned evolutionary times tau of our ancestral probability layer as a text file. Likewise we added command line options to support plotting the consensus sequence logo and a graph representation of the HMM which was previously only possible with the accompanying Jupyter notebook. These changes are currently only available via github (main branch) but will be pushed to pip and conda with the next minor release.

Reviewer #2: Q4. Were SP/TC scores computed for match states only ? since MSA tools do not 'exclude' inserts, learnMSAs alignments might be being unfairly penalised in the SP/TC evaluations.

Authors: The scores are computed for all residues independent of whether the model classifies them as matches or not. Indeed, this is potentially a bias against our method when compared to traditional aligners. However, we believe the score should not depend on a subjective choice of the assessed method of whether something is suitable to be scored or not. Some, but not all, of our benchmark datasets included upper/lower case amino acids objectively indicating conservative regions, but it seemed inconsistent to evaluate them differently than other datasets that lack the distinction.

In addition to SP/TC scores, we also computed the column score (not included in the manuscript) which is a weighted TC score where each reference column is weighted by the number of pairs (excluding gaps). This implicitly favors conserved columns which correspond to the match states in the HMM (assuming it is correct), or put differently errors made when not aligning insertions at all weight very little. However, we saw no noticeable advantage of learnMSA when evaluating under the column score compared to TC score which could indicate that the unfair penalisation is not critical.

Reviewer #2: Q5. You discuss the extension to ensemble/multi-pHMM learning - is this mathematically feasible with the current approach without a grid search to find the optimal number of learned-pHMM models that can describe all sequences ?

Authors: There are several approaches of how at least local alternatives could be used and trained and in doing so the strong assumption that the Markov property constitutes in a standard pHMM could be relaxed. One possibility are different “branches” in a single, global model introduced by new learnable transitions between non-adjacent matches. This could in principle learn an optimal number of sub-models automatically. Feasibility problems might occur when decoding an alignment from such a model. We have not done substantial experiments on this matter yet.

Reviewer #2: Q6. Your point about the weakness of the HomFam dataset is interesting - have any others attempted to correct for this weakness ?

Authors: Not that we know of. But quite the contrary, the recently proposed regressive strategy [Garriga et al, Nature Biotechnology, 2019] might implicitly exploit it by choosing the longest sequence as representative of a cluster and consequently aligning the longest sequences in a dataset first. An

indicator of this is the lower relative performance of regressive T-Coffee on BaliFam compared to HomFam in our experiments.

Reviewer #2: Q7. You note that transformers/etc are complimentary to the learnMSA approach - could grammar based models be employed as priors to increase convergence ?

Authors: This is a good idea but probably out of our scope. We plan to incorporate ideas from Natural Language Processing, which are already explored by others (e.g. Elnaggar, Ahmed, et al. "ProtTrans: towards cracking the language of Life's code through self-supervised deep learning and high performance computing." arXiv preprint arXiv:2007.06225 (2020).)

Reviewer #2: Suggested revisions.
R1. I am not convinced the manuscript supports the abstract's final statement "statistical counter-intuition that more data leads to lower accuracy", and suggest that is reworded to better reflect learnMSAs contribution to the field.

Specifically - most modern MSA tools take advantage of the observation that random sampling leads to a 'good enough' scaffold for constructing an alignment, and alignment errors introduced during heuristics tend to be reduced through the use of pHMMs for realignment. I support the authors demonstration that learnMSA provides a vastly more scalable alternative to 'optimal progressive alignment' (e.g. as implemented in early approaches such as the AMPS toolchain), but the statement that 'more sequences leads to less accurate MSAs' is in my experience not widely recognised the main barrier preventing the construction of MSAs for very large sets of sequences (as opposed to massive datasets in the context of other fields such as proteomics, where the 'chinese restaurant process' needs taking into consideration when attempting to statistically assess low abundance signals). Whilst there are commonalities between individual variation (e.g. species specific insertions, variable repeat regions, rearranged domains, etc), MSA methods tend to handle these by excessive gap insertion rather than erroneous alignment. In this regard, I applaud the authors in their devising of learnMSA's boundary conditions and model surgery heuristics, which I found to be highly effective in separating alignable fron unalignable regions.

Authors: We performed an additional experiment and added a new Figure 3 to the manuscript to support the claim that adding more homologs leads for several popular aligners to a decrease in accuracy. To clearly state this, the fixed reference set of sequences on which the MSA is evaluated is thereby unknown to the aligner. This harmful effect of more data to MSA accuracy has been observed earlier in [Garriga et al, Nature Biotechnology, 2019] (Figure 2) or in [Sievers et al., Molecular Systems Biology, 2011] (Figure 3) and is also the main motivation for recent papers such as [Smirnov, PLoS Computational Biology, 2021]. The new Figure 3 confirms this counterintuitive loss in accuracy with T-Coffee and MAFFT and demonstrates that learnMSA apparently does not suffer from it.

Indeed, aligning a sample subset may be an option to avoid a loss of accuracy when the addition of further sequences decreases the accuracy of the MSA projected to the subset. However, this does not work in the benchmark setting that we followed.
In addition, we changed the wording in the abstract to: “Our results show that learnMSA does not share the counter-intuitive drawback of many popular heuristic aligners which can substantially lose accuracy when many additional homologs are input.”

Reviewer #2: R2. In the opening paragraph early experiments with training pHMMs involved 'hand-holding' - this doesn't really mean anything to the general reader so it should be more fully explained.

Authors: We added an explanation to the manuscript.

Reviewer #2: R3. The authors mention in the introduction that 'common problems are local optima in the parameter space'. No mention is made specifically of how learnMSA avoids this ? In the same spirit, it seems a drastic leap to suggest that statistical learning 'presents itself as a valid approach' in the light of the problems that must be overcome: instead, perhaps acknowledge that if these could be overcome, statistical learning offers a route for computing (ultra-)large MSAs.

Authors: We reformulated this part to avoid the misunderstanding that local optima can be avoided with gradient-based optimization.

Reviewer #2: R4. Method
i. The authors 'Note that pHMM methods can indicate the difference between conserved residues and insertions explicitly' - whilst useful to communicate this distinction, it seems to not follow from the previous sentence (discussing the data-dependent entry- and exit- probabilities) - if there's a clear connection between these statements it would help to clearly explain here.

Authors: We removed the statement as indeed it was not in the right context.

Reviewer #2: ii. The sentence in the paragraph describing explicitly how sequences are padded with terminal symbols could be omitted - this seems an implementation detail (albeit an essential one for the consistency of the system).

Authors: Agreed and deleted.

Reviewer #2: iii. "However, with automatic differentiation learnMSA can make use of the advancing gradient-based optimization toolbox for machine learning
problems." - this looks like it deserves a reference for automatic differentiation (or a review of recent advances in gradient based optimisation)

Authors: We now give such a reference.

Reviewer #2: iv. Recommend adding a few sentences at the start of the 'Training' section to overview the objective of training (multilayer pHMM including ancestral probabilities), and then introduce the naive approach of maximising log likelihood of a random batch.

Authors: We changed it accordingly.

Reviewer #2: v. "For each
possible choice of p and alpha the logarithmic prior densities are
(alpha - 1) ln p + (alpha' - 1) ln (1 - p), where we set alpha' = 1." - is this correct ? if so, what use is alpha' ?

Authors: The larger alpha, the more does the loss function favor large values of p. In theory, we could have chosen any differentiable function for regularization, but we intended not to lose the probabilistic interpretation. Our approach has a theoretical foundation on Dirichlet priors with densities as given by the formula in your question with 2 hyperparameters alpha and alpha’ for each p which have to be chosen appropriately.

Our choice to set alpha’=1 was an ad hoc decision motivated merely by a simplification of the computation and in order to search only a single parameter alpha instead of two. This choice worked, but is of course not necessarily the best. In general, the larger alpha’ the more are large values of (1-p) favored. The sum of both alphas controls the concentration of the density on a single point.

Reviewer #2: R5.Evaluation
i. Figure 3 - I recommend marking TTK_HUMAN as the reference sequence (https://www.jalview.org/help/html/calculations/referenceseq.html) and include the alignment ruler in each MSA visualisation - this may make it easier to find and compare the columns containing each reference sequence position in the alignments produced by each method.

Authors: We changed the figure accordingly and agree that it is now easier to interpret.

Reviewer #2: R6. Conclusions
i. "By design, learnMSA can incorporate any type of sequence context encoded into the HMM alphabet, relaxing the assumption that sites are independent." - for clarity, I recommend you say 'adjacent sites' here, since the pHMM model only explicitly learns transition chains along sequences, rather than long-range covariation.

Authors: We improved this paragraph in manuscript.

Reviewer #2: Grammar & Typos [...]

Authors: Thank you. We made the changes.

Close
