# Supplementary material for: learnMSA: learning and aligning large protein families
Source: Gigascience. 2022 Nov 18;11:giac104. doi: 10.1093/gigascience/giac104 (PMC9673500; doi:10.1093/gigascience/giac104)
Supplement: giac104_Response_to_Reviewer_Comments_Revision_1 [file giac104_response_to_reviewer_comments_revision_1.pdf]

# Author's Response To Reviewer Comments

Close

We added the citation to the GigaDB dataset.

Close
