# Supplementary material for: learnMSA: learning and aligning large protein families
Source: Gigascience. 2022 Nov 18;11:giac104. doi: 10.1093/gigascience/giac104 (PMC9673500; doi:10.1093/gigascience/giac104)
Supplement: giac104_Reviewer_1_Report_Original_Submission — Julie Thompson -- 7/10/2022 Reviewed [file giac104_reviewer_1_report_original_submission.pdf]

Reviewer Report

Title: learnMSA: Learning and Aligning Large Protein Families

Version: Original Submission **Date:** 7/10/2022

Reviewer name: Julie Thompson

Reviewer Comments to Author:

The article describes an original method, learnMSA, for construction of large multiple sequence alignments, that uses a recurrent neural network approach to learn profile Hidden Markov models of protein sequence families. The method is evaluated, and compared to state of the art methods, on existing benchmarks containing very large test sets some with more than a million sequences. The methods and evaluation experiments are clearly described and the results indicate that learnMSA is competitive in terms of alignment accuracy and calculation time.
Major criticisms:
1. Other recent work using deep learning approaches to construct multiple sequence alignments should be discussed, and if possible included in the comparisons. For example, Zhang et al. DeepMSA: constructing deep multiple sequence alignment to improve contact prediction and fold-recognition for distant-homology proteins. Bioinformatics. 2020; Kuang et al. DLPAlign: A Deep Learning based Progressive Alignment Method for Multiple Protein Sequences CSBio2020: CSBio '20: Proceedings of the Eleventh International Conference on Computational Systems-Biology and Bioinformatics; Jafari et al. Using deep reinforcement learning approach for solving the multiple sequence alignment problem. SN Applied Sciences volume 1, Article number: 592 (2019)
2. I tried to install and run the software (using Tensorflow 2.5.0), but it failed with the following error message: "NotImplementedError: Cannot convert a symbolic Tensor (msa_hmm_layer/strided_slice_47:0) to a numpy array. This error may indicate that you're trying to pass a Tensor to a NumPy call, which is not supported".
More minor comments:
1. The method is demonstrated using protein sequences. Is it also suitable for the alignment of DNA/RNA sequences?

Level of Interest

Please indicate how interesting you found the manuscript: Choose an item.

Quality of Written English

Please indicate the quality of language in the manuscript: Choose an item.

Declaration of Competing Interests

Please complete a declaration of competing interests, considering the following questions:

- Have you in the past five years received reimbursements, fees, funding, or salary from an organisation that may in any way gain or lose financially from the publication of this manuscript, either now or in the future?
- Do you hold any stocks or shares in an organisation that may in any way gain or lose financially from the publication of this manuscript, either now or in the future?
- Do you hold or are you currently applying for any patents relating to the content of the manuscript?
- Have you received reimbursements, fees, funding, or salary from an organization that holds or has applied for patents relating to the content of the manuscript?
- Do you have any other financial competing interests?
- Do you have any non-financial competing interests in relation to this paper?

If you can answer no to all of the above, write 'I declare that I have no competing interests' below. If your reply is yes to any, please give details below.

I declare that I have no competing interests

I agree to the open peer review policy of the journal. I understand that my name will be included on my report to the authors and, if the manuscript is accepted for publication, my named report including any attachments I upload will be posted on the website along with the authors' responses. I agree for my report to be made available under an Open Access Creative Commons CC-BY license (http://creativecommons.org/licenses/by/4.0/). I understand that any comments which I do not wish to be included in my named report can be included as confidential comments to the editors, which will not be published.

Choose an item.

To further support our reviewers, we have joined with Publons, where you can gain additional credit to further highlight your hard work (see: https://publons.com/journal/530/gigascience). On publication of this paper, your review will be automatically added to Publons, you can then choose whether or not to claim your Publons credit. I understand this statement.

Yes Choose an item.
