# Supplementary material for: learnMSA: learning and aligning large protein families
Source: Gigascience. 2022 Nov 18;11:giac104. doi: 10.1093/gigascience/giac104 (PMC9673500; doi:10.1093/gigascience/giac104)
Supplement: giac104_Reviewer_2_Report_Original_Submission — James Procter -- 7/13/2022 Reviewed [file giac104_reviewer_2_report_original_submission.pdf]

Reviewer Report

Title: learnMSA: Learning and Aligning Large Protein Families

Version: Original Submission **Date:** 7/13/2022

Reviewer name: James Procter

Reviewer Comments to Author:

The authors present a practically applicable implementation of a hitherto unexplored approach for the multiple sequence alignment problem that was first described in the 1990's by Eddy, Krogh, Sjolander, et al.. learnMSA takes advantage of tensorflow to perform 'statistical alignment' by iterations of steepest descent and algorithmic pruning to identify a single optimal profile hidden markov model for a set of sequences. The pHMM model advances Eddy's Plan7 architecture in its support for both ancestral state and repeat regions, and the accompanying code provides mechanisms for HMM model visualisation in addition to emission of the multiple alignment of the given sequences induced by the final model.

Code. The authors provide a repository containing a python module (with tests), a python script for command line execution, and a jupyter notebook demonstrating the methodology and results visualisation. Whilst documentation is sparse, the code performs as described. I look forward to the package being made available via pip and ultimately bioconda. I also look forward to enhancements made by the authors and the future learnMSA community that enable users to make use of the additional data embodied by the learned pHMM.

Manuscript. Overall, the manuscript presents a clear account of the theoretical approach and practical implementation. Clarity could be improved in some areas, and suggestions are made below. The authors also devised challenging benchmarks in order to evaluate their method, which demonstrated both its strengths and potential weaknesses. Whilst the results are convincing, they necessarily rely on MSA statistics that are difficult to interpret, but this should not be a barrier to publication. Ideally, a more robust analysis could be performed with gold standard data such as structures, perhaps by adapting established MSA benchmarking tools such as OxBench.

Below I note a number of questions for the authors, followed by suggested revisions, and finally a handful of grammar/typo fixes.

Q1. are the disadvantages regarding domain repeats (in Viterbi decoding) addressable ?

Q2. the model surgery employs a 50% threshold for discard of underpopulated match states or over-represented insertions - are there situations where this could cycle ? If so, can such pathologies be detected in the reported statistics for the model ? Could these heuristics also cause the problems when aligning sequences of greatly differing lengths ?

Q3. The command line tool only supports output of the final MSA - is there utility in a) reporting also the pHMM for the MSA and b) the ancestral probabilities ?

Q4. Were SP/TC scores computed for match states only ? since MSA tools do not 'exclude' inserts, learnMSAs alignments might be being unfairly penalised in the SP/TC evaluations.

Q5. You discuss the extension to ensemble/multi-pHMM learning - is this mathematically feasible with

the current approach without a grid search to find the optimal number of learned-pHMM models that can describe all sequences ?
Q6. Your point about the weakness of the HomFam dataset is interesting - have any others attempted to correct for this weakness ?
Q7. You note that transformers/etc are complimentary to the learnMSA approach - could grammar based models be employed as priors to increase convergence ?
Suggested revisions.
R1. I am not convinced the manuscript supports the abstract's final statement "statistical counter-intuition that more data leads to lower accuracy", and suggest that is reworded to better reflect learnMSAs contribution to the field.
Specifically - most modern MSA tools take advantage of the observation that random sampling leads to a 'good enough' scaffold for constructing an alignment, and alignment errors introduced during heuristics tend to be reduced through the use of pHMMs for realignment. I support the authors demonstration that learnMSA provides a vastly more scalable alternative to 'optimal progressive alignment' (e.g. as implemented in early approaches such as the AMPS toolchain), but the statement that 'more sequences leads to less accurate MSAs' is in my experience not widely recognised the main barrier preventing the construction of MSAs for very large sets of sequences (as opposed to massive datasets in the context of other fields such as proteomics, where the 'chinese restaurant process' needs taking into consideration when attempting to statistically assess low abundance signals). Whilst there are commonalities between individual variation (e.g. species specific insertions, variable repeat regions, rearranged domains, etc), MSA methods tend to handle these by excessive gap insertion rather than erroneous alignment. In this regard, I applaud the authors in their devising of learnMSA's boundary conditions and model surgery heuristics, which I found to be highly effective in separating alignable fron unalignable regions.
R2. In the opening paragraph early experiments with training pHMMs involved 'hand-holding' - this doesn't really mean anything to the general reader so it should be more fully explained.
R3. The authors mention in the introduction that 'common problems are local optima in the parameter space'. No mention is made specifically of how learnMSA avoids this ? In the same spirit, it seems a drastic leap to suggest that statistical learning 'presents itself as a valid approach' in the light of the problems that must be overcome: instead, perhaps acknowledge that if these could be overcome, statistical learning offers a route for computing (ultra-)large MSAs.
R4. Method
i. The authors 'Note that pHMM methods can indicate the difference between conserved residues and insertions explicitly' - whilst useful to communicate this distinction, it seems to not follow from the previous sentence (discussing the data-dependent entry- and exit- probabilities) - if there's a clear connection between these statements it would help to clearly explain here.
ii. The sentence in the paragraph describing explicitly how sequences are padded with terminal symbols could be omitted - this seems an implementation detail (albeit an essential one for the consistency of the system).
iii. "However, with automatic
differentiation learnMSA can make use of the advancing
gradient-based optimization toolbox for machine learning

problems." - this looks like it deserves a reference for automatic differentiation (or a review of recent advances in gradient based optimisation)
iv. Recommend adding a few sentences at the start of the 'Training' section to overview the objective of training (multilayer pHMM including ancestral probabilities), and then introduce the naive approach of maximising log likelihood of a random batch.
v. "For each
possible choice of p and â€€alpha the logarithmic prior densities are
(alphaâ€€ - 1) ln p + (â€€alpha' - 1) ln (1 - p), where we set â€€alpha' = 1." - is this correct ? if so, what use is alpha' ?
R5.Evaluation
i. Figure 3 - I recommend marking TTK_HUMAN as the reference sequence (https://www.jalview.org/help/html/calculations/referenceseq.html) and include the alignment ruler in each MSA visualisation - this may make it easier to find and compare the columns containing each reference sequence position in the alignments produced by each method.
R6. Conclusions
i. "By design, learnMSA can incorporate any type of sequence context encoded into the HMM alphabet, relaxing the assumption that sites are independent." - for clarity, I recommend you say 'adjacent sites' here, since the pHMM model only explicitly learns transition chains along sequences, rather than long-range covariation.
Grammar & Typos
"The likelihood be efficiently computed
with dynamic programming using either the forward- or the
backward algorithm [19]" - suggest 'likelihood can be efficiently computed'
"we refer for Viterbi to the extensive
literature." - should that be 'we refer the reader to the extensive literature' ? presumably the previous sentence provides the canonical reference for viterbi - is this sentence necessary ?
"We found
that c = 0.8 works good. " - works 'well' ?
"It should be pointed out, that learnMSAs is highly more accurate than other methods
when aligning families that contain multihits" - suggest remove 'highly'

Level of Interest

Please indicate how interesting you found the manuscript: Choose an item.

Quality of Written English

Please indicate the quality of language in the manuscript: Choose an item.

Declaration of Competing Interests

Please complete a declaration of competing interests, considering the following questions:

- Have you in the past five years received reimbursements, fees, funding, or salary from an organisation that may in any way gain or lose financially from the publication of this manuscript, either now or in the future?
- Do you hold any stocks or shares in an organisation that may in any way gain or lose financially from the publication of this manuscript, either now or in the future?
- Do you hold or are you currently applying for any patents relating to the content of the manuscript?
- Have you received reimbursements, fees, funding, or salary from an organization that holds or has applied for patents relating to the content of the manuscript?
- Do you have any other financial competing interests?
- Do you have any non-financial competing interests in relation to this paper?

If you can answer no to all of the above, write 'I declare that I have no competing interests' below. If your reply is yes to any, please give details below.

I declare that I have no competing interests

I agree to the open peer review policy of the journal. I understand that my name will be included on my report to the authors and, if the manuscript is accepted for publication, my named report including any attachments I upload will be posted on the website along with the authors' responses. I agree for my report to be made available under an Open Access Creative Commons CC-BY license (http://creativecommons.org/licenses/by/4.0/). I understand that any comments which I do not wish to be included in my named report can be included as confidential comments to the editors, which will not be published.

Choose an item.

To further support our reviewers, we have joined with Publons, where you can gain additional credit to further highlight your hard work (see: https://publons.com/journal/530/gigascience). On publication of this paper, your review will be automatically added to Publons, you can then choose whether or not to claim your Publons credit. I understand this statement.

Yes Choose an item.
